# Dual‐function, Reusable, and Flexible Thermal Interface for Kinetic Monitoring of In Vitro Bioassays

**DOI:** 10.1002/smtd.202501243

**Published:** 2025-12-18

**Authors:** Daniel Nieder, Isli Cela, Željko Janićijević, Xinne Zhao, Larysa Baraban

**Affiliations:** ^1^ Institute of Radiopharmaceutical Cancer Research Helmholtz‐Zentrum Dresden‐Rossendorf (HZDR) Bautzner Landstraße 400 01328 Dresden Germany; ^2^ Else Kröner Fresenius Center for Digital Health, Faculty of Medicine Carl Gustav Carus Technische Universität Dresden 01062 Dresden Germany

**Keywords:** bioassay, biosensor, *E. coli*, thermal effusivity, thermal sensing interface

## Abstract

Kinetic monitoring in life sciences is predominantly performed using contactless optical techniques. Miniaturized electronic sensing alternatives typically require direct contact with the sample. We introduce a dual‐function thermal actuator/sensor that uniquely combines microwell‐independent temperature control of a modified microplate and simultaneous measurement of real‐time changes in thermal effusivity, offering both electronic readout and contactless sensing. The performance is demonstrated by monitoring Escherichia coli (*E. coli*) growth and assessing the effect of cefotaxime (CTX) as a use‐case application, benchmarking it against state‐of‐the‐art optical techniques. By qualitative comparison of characteristic data features, we report that the thermal sensing modality showed an increased response signal compared to optical density (OD) measurements in interface‐dominated processes, which can be observed under experimental conditions where metabolic or morphological adaptation happens in response to CTX. Additionally, we developed an autonomous full curve data analysis approach for modified Transient Plane Source (mTPS) data, offering high robustness and lower susceptibility to systematic errors and bias. Our technique emphasizes interface‐dominant processes, thereby complementing the assessment of bulk properties obtained by traditional optical techniques. The developed thermal interface is reusable, highly integrable, low‐cost, easy to use, non‐contact, and label‐free, offering a versatile platform for bioassay development and dynamic biological studies.

## Graphical abstract

This work presents a reusable, dual‐function thermal interface for non‐contact, label‐free sensing and microwell‐independent temperature control in microplates within a single element. Real‐time monitoring of *E. coli* growth and the response to antibiotic treatment demonstrates the sensor's enhanced sensitivity in interface‐dominated processes compared to optical techniques. This method provides a powerful complement to the kinetic monitoring landscape in life sciences.

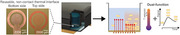

## 1 Introduction

Monitoring of bacterial proliferation is essential across diverse fields, including healthcare,^[^ ^1^ ^]^ water quality management,^[^ ^2^ ^]^ agriculture,^[^ ^3^ ^]^ and food safety.^[^ ^4^ ^]^ Rapid and accurate detection of bacterial growth plays a critical role in diagnosing infections, preventing contamination, and optimizing antimicrobial treatments. Microplates are a gold standard for high‐throughput bioassays due to their versatility and scalability, with proliferation assays being widely used to evaluate bacterial growth dynamics and responses to external stimuli.^[^ ^5^, ^6^, ^7^ ^]^ Conventional microplate readers rely dominantly on optical detection methods, such as absorbance, fluorescence, and luminescence, which provide robust measurements but typically require labels or dyes.^[^ ^8^ ^]^ Despite their utility, these commonly used methods can be destructive, limited to endpoint analysis, affected by photobleaching, and require complex sample preparation.^[^ ^9^ ^]^ In microbial bioassays, changes in cell morphology, such as filamentation or swelling, can significantly affect optical readouts, as these altered shapes scatter light differently than typical rod‐shaped bacteria. As a result, the optical density (OD) may not accurately represent the true number of viable bacteria.^[^ ^10^ ^]^ In OD measurements, the signal describes the attenuation in the intensity of a radiation beam passing through a macroscopically homogeneous medium with which it interacts, and, therefore, is more sensitive to phenomena happening in bulk, rather than at the interface.^[^ ^11^ ^]^ Nevertheless, many microbiological assays and phenomena take place at or near interfaces (e.g., cell culture on agar plates or biofilm formation at the bottom of static microplate wells). This mismatch between what is measured (bulk) and where biological activity often occurs (interface) highlights a limitation of conventional methods and motivates the development of sensing techniques with enhanced surface sensitivity.

Various types of miniaturized electronic sensors (such as field‐effect transistor‐based, capacitive, piezoelectric, or impedance‐based) have emerged as promising alternatives for label‐free, real‐time monitoring of microbial cultures.^[^ ^12^, ^13^, ^14^, ^15^, ^16^ ^]^ For example, by embedding electrodes in microplates, electrochemical impedance spectroscopy (EIS) continuously measures electrical impedance changes correlating with bacterial proliferation.^[^ ^17^ ^]^ Despite multiple advantages, including minimal sample preparation and continuous monitoring capabilities, EIS requires an electrode interface in direct contact with the sample, and, in turn, remains susceptible to interference caused by medium composition and ionic fluctuations.

Beyond optical and electrical methods, microfluidic formats such as Micro Total Analysis Systems (µTAS) and Lab‐on‐Chip platforms offer advanced bacterial monitoring by reducing reagent consumption and enabling compact, high‐throughput screening. However, the widespread adoption and clinical implementation of these systems remain limited by fabrication complexity, specialized instrumentation, and maintenance challenges.^[^ ^18^, ^19^, ^20^ ^]^ Therefore, there is a need to develop continuous monitoring methods that offer easy handling, non‐contact readout, reduced dependence on the medium properties, and compatibility with miniaturization technologies.

A largely overlooked but highly promising sensing approach is thermal monitoring, which exploits changes in the thermal properties of a sample as an indirect but sensitive indicator of biological processes.^[^ ^21^ ^]^ Thermal sensing has previously been applied in calorimetric and heat transfer studies, but its potential for bioassays remains underexplored.^[^ ^22^, ^23^ ^]^ Among various thermal techniques, the modified Transient Plane Source (mTPS) method stands out for its simplicity, non‐contact nature, and ease of integration into existing assay platforms.^[^ ^17^, ^24^, ^25^ ^]^ This method was originally developed for the physical characterization of materials, including solids,^[^ ^24^, ^26^ ^]^ powders,^[^ ^27^ ^]^ and liquids.^[^ ^14^, ^28^ ^]^ Recent advancements have demonstrated the feasibility of mTPS for monitoring biological systems, where yeast cell growth and metabolic activity have been measured in the time domain.^[^ ^17^, ^29^ ^]^ So far, for mTPS studies, the temperature was either uncontrolled or externally controlled via separate and bulky temperature control systems, such as cell incubators.

Here, we introduce a novel thermal sensing principle that enables simultaneous bacterial monitoring and independent temperature control within microplate wells. By leveraging mTPS technology, we establish a reusable, flexible, and noninvasive dual‐function thermal interface capable of both real‐time kinetic sensing and localized thermal regulation. The sensing relies on the detection of changes in thermal effusivity (*e*) of the sample, a physical property that describes the exchange of thermal energy with its surroundings, particularly when in contact with another material at a different temperature.^[^ ^30^ ^]^ The fundamental principles of the mTPS technique for life science applications have been previously described in the literature, where it is used for measuring organic solvents,^[^ ^14^ ^]^ fruit juices,^[^ ^14^ ^]^ and yeast cell growth.^[^ ^21^, ^29^ ^]^ Unlike conventional methods, our approach is entirely label‐free, non‐contact, and highly integrable into standard microplate formats, combining the advantages of optical and impedance‐based methods. We demonstrate its application by monitoring the growth dynamics of *Escherichia* *coli* (*E. coli*) under controlled conditions and assessing antibiotic‐induced changes in bacterial proliferation. Distinct thermal response patterns could be classified, which helped to assess morphological and metabolic adaptation due to the cefotaxime (CTX) stimulus. The thermal interface provides enhanced information when monitoring surface‐dominant biological processes and shows performance comparable to conventional methods in bulk‐dominated assays. This study highlights the transformative potential of thermal biosensing as a new paradigm in kinetic bioassays. By offering a cost‐effective, reusable, non‐contact, scalable, and easily implementable alternative to existing technologies, our approach paves the way for more versatile and accessible real‐time bacterial monitoring in both research and industrial settings.

## 2 Thermal Interface Sensing

**Figure 1:**
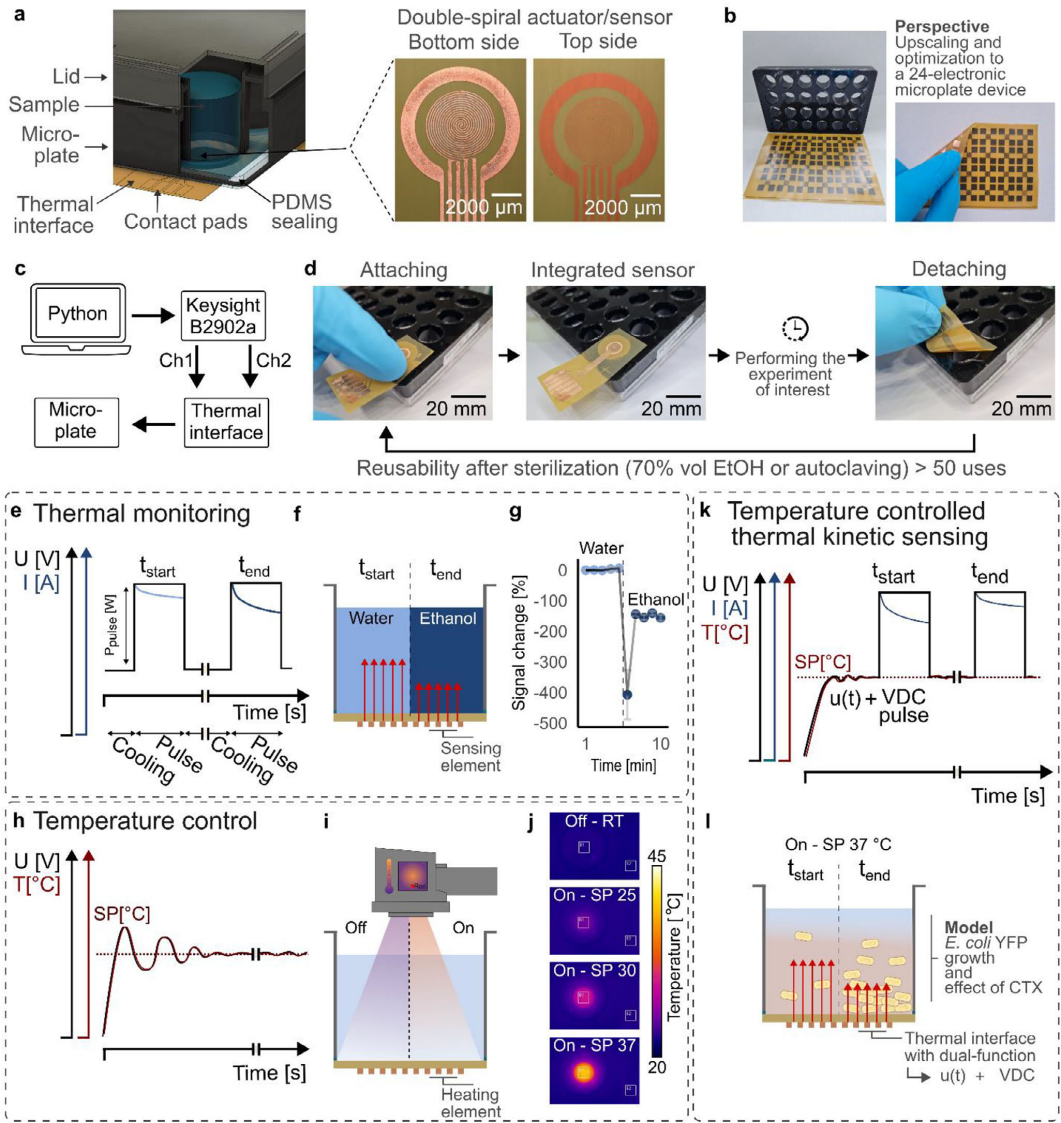
a) Schematic illustration of the in‐house modified microplate featuring an integrated thermal interface. Microscope images show the top and bottom sides of the interface, with only the polyimide top side in contact with the sample during operation, ensuring biochemical inertness. b) Perspective view illustrating the concept of an electronic microplate, highlighting the scalability and seamless integration into existing workflows. The 24 thermal interfaces were fabricated in‐house on a single polyimide sheet. c) Block diagram of the experimental setup, where a custom‐made Python script controls the operation of the SMU (Source Measure Unit). d) Photographs demonstrating straightforward and reusable integration of the flexible thermal interface into the microplate. e) Modified Transient Plane Source sensing principle, where an inversely proportional current drop occurs when applying a short DC heating pulse. f) Liquid model illustrating the real‐time sensor response to varying thermal effusivity, achieved by changing the liquid in the well. g) Real‐time signal response showing the sensor's reaction to liquid exchange. h) Temperature control concept using a PID‐control feedback loop, ensuring precise temperature oscillation around the setpoint via continuous voltage adjustments on the thermal element. i) Experimental setup for assessing the temperature control capability, captured by an infrared thermal camera. j) Thermal camera images showing different temperature setpoints, including PID‐off, 25, 30, and 37 °C. k) Schematic illustration of the combined dual functionality, enabling continuous thermal effusivity sensing and temperature control. The dual functionality is coordinated by a customized Python script controlling the SMU. l) Schematic representation of the setup used for *E. coli* incubation and growth monitoring.

In this study, we extend the functionality of mTPS by integrating temperature control, allowing simultaneous *e* sensing and temperature regulation within a single element. This approach allows us to monitor bacterial growth while maintaining a precise bioassay temperature in each microplate well. The corresponding thermal interface is visualized in **Figure** **1**a. One of the key features of the thermal interface is that the double‐spiral element is operated in non‐contact mode, which means that the sensing element stays at the opposite side of the substrate, while only the top side of the thermal interface (bare polyimide) is in contact with the sample. This ensures high biocompatibility, no corrosion, and no fouling.

The mTPS technique works by applying a short direct current (DC) voltage pulse to the sensor element, generating Joule heating, which is then used to measure the *e* of the system (see Figure 1e,f). The thermal properties of the sample are inferred from the inversely proportional feedback in current, as changes in the thermal environment affect the heat dissipation through the sensor. The duration and power of the heating pulses can be tuned based on experimental requirements, typically ranging from 0.5 to 2 s and between 10 and 100 mW, respectively.^[^ ^14^, ^21^, ^25^ ^]^ To prevent overheating and ensure accurate measurements, thermal pulses are applied at intervals of tens of seconds to minutes, allowing the sample to return to its baseline temperature between pulses.

In this work, we introduce an additional functionality: temperature control between the heating pulses by adjusting the current flow through the thermal element. By fine‐tuning the power delivered to the system, we regulate the heat generated through Joule heating (see Figure 1h). This allows us to maintain a stable temperature in the microplate wells, crucial for ensuring the optimal growth environment for bacterial cultures, such as keeping *E. coli* YFP at 37 °C.

Temperature control is achieved through continuous resistance measurements of the double‐spiral element. At the start of each experiment, the initial resistance of the sensor is measured at room temperature (RT). Given the linear relationship between resistance and temperature in the relevant range, the temperature of the sensor can be continuously calculated throughout the experiment by monitoring the resistance. This forms the basis for precise temperature control during the measurement process.

To achieve stable temperature regulation, a Proportional‐Integral‐Derivative (PID) control system is programmed. The PID controller adjusts the current flow to the sensor element based on real‐time feedback from the resistance measurements, ensuring that the temperature remains close to the setpoint (Figure S1, Supporting Information). The proportional component of the PID system adjusts the current in response to the immediate difference between the measured and desired temperatures. The integral component accounts for cumulative temperature deviations over time, while the derivative component anticipates future changes by assessing the rate of temperature variation. Together, these factors enable smooth and stable temperature control, minimizing overshooting or oscillations that could affect biological processes.

This dual functionality of the thermal interface, combining real‐time measurement of thermal properties and precise temperature control, enhances its applicability in bioassays (see Figure 1k,l). In addition to the monitoring of changes in *e* due to bacterial growth, the system provides a stable environment that can be independently controlled in each well. This enables more reliable experiments, where temperature fluctuations are minimized, and biological responses can be accurately assessed in real time.

## 3 Results and Discussion

### 3.1 Integrated, Reusable Thermal Interface for Localized Heating and Real‐Time Effusivity Sensing

We present a reusable, noninvasive thermal interface that integrates seamlessly into commercially available 24‐well microplates, enabling simultaneous temperature control and real‐time *e* sensing. The sensor is fabricated on a 25 µm‐thick flexible polyimide substrate and features a double‐spiral thermal element optimized for localized thermal interaction at the interface (see Figure 1a; Figure S3, Supporting Information). Integration into bottomless microplates is achieved using a polydimethylsiloxane (PDMS) sealing layer, ensuring a tight, leak‐free connection that is fully compatible with standard laboratory workflows (see Figure 1d; Figure S3, Supporting Information). All components are sterilizable, allowing for repeated use across multiple experiments. Over six months of use, including more than 50 experiments, the microplate maintained consistent performance with no degradation. Only four thermal interfaces were required in total, underscoring their long‐term reusability. The non‐contact design ensures that only the inert substrate (polyimide) is exposed to the sample, minimizing fouling and corrosion, which is an advantage over conventional techniques like EIS relying on direct electrode contact. For further information on the thermal interface geometry and the setup architecture, refer to Note S1 (Supporting Information).

Temperature control is implemented via a PID feedback system, optimized for rapid and stable thermal regulation (Figure S1, Supporting Information). Stable temperatures across the range of 25–40 °C were reached within 60 s with an accuracy of 99.56 ± 0.37%. Infrared thermography confirmed uniform and precise heating (see Figure 1i,j; Figure S2, Supporting Information). The compact, microwell‐specific heating design allows for energy‐efficient actuation within individual wells, reducing the need for full‐incubator heating and supporting integration into full microplate formats (see perspective shown in Figure 1b). For further information on the temperature control assessment, refer to Note S2 (Supporting Information).

To support robust and reproducible sensing, we implemented an autonomous full‐curve fitting method for analyzing thermal responses. In contrast to conventional approaches, where a manually selected linear interval of the heating curve is used to extract effusivity‐related parameters,^[^ ^17^, ^25^, ^29^ ^]^ our method fits the entire thermal response with a second‐order polynomial of the form *y*  =  *ax* ^2^ + *bx* + *c*. Here, the coefficient *a* reliably captures changes in *e* over time, serving as the primary kinetic marker. This approach eliminates subjective user bias, increases consistency, and enhances sensitivity to dynamic changes, as demonstrated in interface‐specific measurements of biological and physical transitions. Initial heating‐related fluctuations are accounted for by zeroing negative percentage changes during thermal stabilization. For a detailed explanation and validation of this method, refer to Note S3 and Figure S8 (Supporting Information).

Overall, this platform offers a flexible and sustainable alternative to existing contact‐based or single‐use biosensing methods, supporting high‐throughput and real‐time analysis within standard microplate formats.

#### 3.1.1 Thermal Interface in Bacterial Growth and Antibiotics Assays

The capability of real‐time kinetic sensing is demonstrated in Figure 1e,f,g. To evaluate the sensor's response to different thermal properties of the sample inside the well, deionized (DI) water was measured for 5 min before being replaced with ethanol during the cooling phase between pulses 5 and 6. This sudden liquid exchange represents a dynamic shift in thermal properties near the sensor interface. DI water has a *e* of 1530 J (m^2^ K√s)^−1^, while ethanol has a lower *e* of 569 J (m^2^ K√s)^−1^. The mTPS method has previously been shown to be sensitive to changes in *e* in homogeneous liquid samples, as demonstrated in literature^[^ ^14^ ^]^ and also verified for our in‐house fabricated sensors (Figure S7, Supporting Information). The real‐time sensor response shown in Figure 1g confirms the sensor's capability to detect dynamic changes in thermal properties.

The sensors’ ability to detect kinetic changes in *e* is the underlying principle for measuring dynamic changes in biological cultures. The principle is schematically shown in Figure 1l, where a change in biomass, e.g., cell growth or cell death, changes the *e* of the sample. Before analyzing the data in detail, it is essential to emphasize the key differences between the new thermal interface and OD measurements, as this distinction is crucial for positioning *e* sensing within the landscape of kinetic sensing. State‐of‐the‐art optical techniques, such as OD measurements in a microplate reader, rely on light absorption and scattering to quantify bacterial growth. These effects are measured across the entire volume of the sample, providing a bulk average of cell concentration. In contrast, the thermal interface is governed by the probing depth of the heating pulse, making it highly sensitive to changes in physical properties near the sensor surface rather than the entire volume. The active sensing area in microplate readers refers to the specific spot within a microwell where the light beam passes through the entire height of the sample. This area is typically defined by the optical path between the light source and the detector. In the thermal interface, the active area is defined by the geometry of the sensing element. The heating pulse can be approximated to be 1D, and therefore, the active area is equal to the surface area of the double‐spiral element (17.35 mm^2^). The probing depth of the thermal measurement depends on factors such as pulse power, duration, and sample composition, and can be approximated using: $d\approx \sqrt{\alpha t}$, where *α* is the thermal diffusivity, and *t* is the heating time.^[^ ^31^ ^]^ Using this equation, the probing depth is estimated to be ≈500 µm for biological samples in aqueous solution. In OD measurements, the probing depth is defined as the distance the light beam travels through the sample solution within the well. Thus, compared to OD, the thermal interface signal captures a larger active surface area but with a smaller penetration depth.

To assess the functionality of the thermal interface, bacterial growth curves were measured under various conditions, including exposure to the bacteriolytic antibiotic CTX.^[^ ^32^ ^]^ The Minimum Inhibitory Concentration (MIC) is the lowest concentration of an antibiotic that inhibits the visible growth of a microorganism and is an important parameter in microbiology as it helps define the effective dosage range for antimicrobial agents. The antibiotic‐sensitive bacterial strain used in this work was *E. coli* YFP (MG1655 galK::SYFP2‐FRT‐*cat*‐FRT), which has a MIC of 0.0625 µg mL^−1^ for CTX in standardized systems. Standardized system in this context refers to experiments performed in commercial devices under established conditions, i.e., a microplate reader with incubation functionality (temperature at 37 °C and shaking at 170 rpm). In a previous study, *E. coli* cultures were incubated at an initial inoculum concentration of OD_600_ = 0.01 A (5.1 × 10^6^ cells mL^−1^) at 37 °C for 24 h, and the MIC was determined based on an OD_600_ > 0.2 A (1.0 × 10^8^ cells mL^−1^).^[^ ^33^ ^]^ However, MIC depends on the experimental settings; therefore, considering factors like inoculum concentration, timepoint of antibiotic addition, and shaker settings can influence the MIC. The MIC for our experimental system and conditions is determined to be 1 < MIC < 10 µg mL^−1^ CTX. The deviation from the literature value can be explained by factors such as inoculum concentration, incubation temperature, shaking speed (or incubation without shaking), culture volume, and timing of antibiotic addition (which is tied to the inoculum concentration).

Throughout the study, 21 sets of different experimental conditions (in terms of CTX spiking concentration and addition time points) were tested. The results can be classified into distinct cases. Case 0 (no CTX addition) and case 1 (CTX < MIC) represent bulk‐dominated biological responses, case 2 (CTX >> MIC) represents complete bacterial lysis, case 3 (CTX > MIC) represents filamentous stress response before complete bacterial lysis, and case 4 (CTX > MIC, but CTX added at later timepoints) represents metabolic adaptation in terms of bacterial persistence. In the context of this work, bulk‐dominated and interface‐dominated processes are distinguished by the effective sensing volume of the thermal measurement, which extends from the solid–liquid boundary into the sample over the probing depth (≈500 µm). This definition encompasses both adherent and sedimented cells, rather than being restricted to a single surface layer. These cases explain specific biological responses to stress. In the following section, we will have a detailed look at the thermal interface response, compare it to state‐of‐the‐art OD measurements, and outline key differences between these techniques, thereby highlighting their complementarity and application‐specific advantages.

### 3.2 Case 0 (no CTX addition) and Case 1 (CTX < MIC): Bulk‐Dominated Events

**Figure 2:**
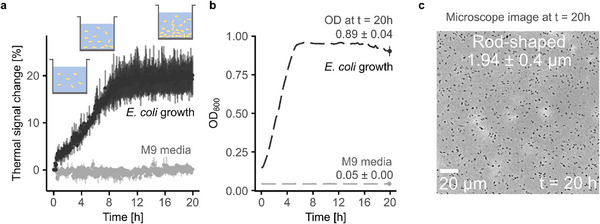
Real‐time measurement of *E. coli* growth recorded using the thermal interface a) and an OD microplate reader b). The temperature was controlled using the programmable heating function of the thermal interface to maintain 37 °C throughout the experiment. The evolution of the *E. coli* culture over time is schematically shown in (a), illustrating growth dynamics under controlled conditions. The plots show mean ± sd from n = 3 repetitions. Cell density = 7.5 × 10^8^ ∙ OD_Plate_ – 1.7 × 10^7^ cells mL^−1^. c) Optical micrograph of *E. coli* culture after 20 h of incubation, showing the final size and morphology of bacteria.

Under all tested conditions, bacterial growth could be classified into distinct response profiles, with case 0 and case 1 representing bulk‐dominated events. In case 0, which serves as the reference condition, *E. coli* was cultivated without the addition of antibiotics. Both the thermal interface and OD measurements captured the exponential growth followed by a transition to the stationary phase (see **Figure** **2**a,b). Growth curve fitting confirmed typical proliferation kinetics (Table S1, Supporting Information), and microscopy after 20 h showed rod‐shaped bacteria of expected length, confirming normal physiological development (see Figure 2c). To quantitatively compare growth trends, we applied curve fitting to both datasets using the growthcurver R package, which allows the extraction of characteristic parameters (see Figure 4b).^[^ ^34^ ^]^ However, it is important to emphasize that these two sensing modalities are not directly comparable. While OD provides a bulk‐averaged signal across the entire well volume, the thermal interface captures a localized, interface‐dominated response. Consequently, extracted kinetic parameters from OD and thermal data differ in both magnitude and dynamics, reflecting the fundamentally different sensing depths and physical principles behind each method.

**Figure 3:**
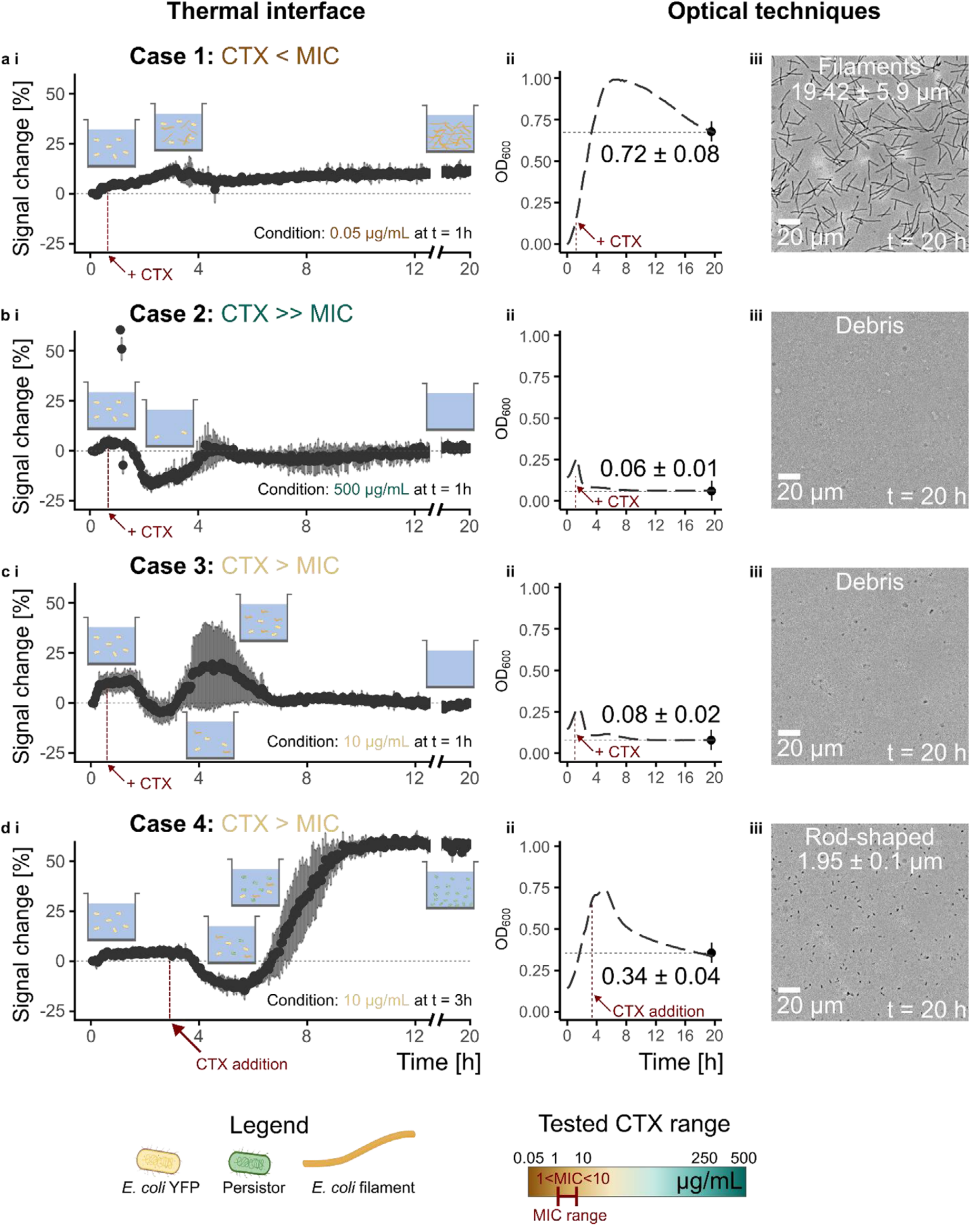
a–d panel i) Real‐time thermal interface data. Observed biological responses can be classified into unique cases. Each case has a unique data trend that can be biologically explained. The biological response is schematically illustrated in panel thermal interface i), featuring *E. coli* growth, filamentation, lysis, and persister cell formation. Case 1 represents the response to sub‐MIC CTX concentration, where the biological response remains bulk‐dominated. In response to CTX, the *E. coli* undergoes filamentation (a i)). Case 2 shows the response to CTX >> MIC, which leads to complete bacterial lysis (b i)). Case 3 represents the sensor's response to CTX > MIC, which results in a filamentous stress response before complete lysis (c i)). Case 4 represents a two‐step response to the induced step, which results in filamentation and metabolic adaptation (persister cell formation) (d i)). The plots show mean ± sd from n = 3 repetitions. a–d panel ii) OD measurements corresponding to the different cases. The plots show mean ± sd from n = 3 repetitions. Cell density = 7.5 × 10^8^ ∙ OD_Plate_ – 1.7 × 10^7^ cells mL^−1^. a–d panel iii) Optical micrographs acquired at the end of the 20 h experiment, revealing the current morphology of *E. coli*.

**Figure 4:**
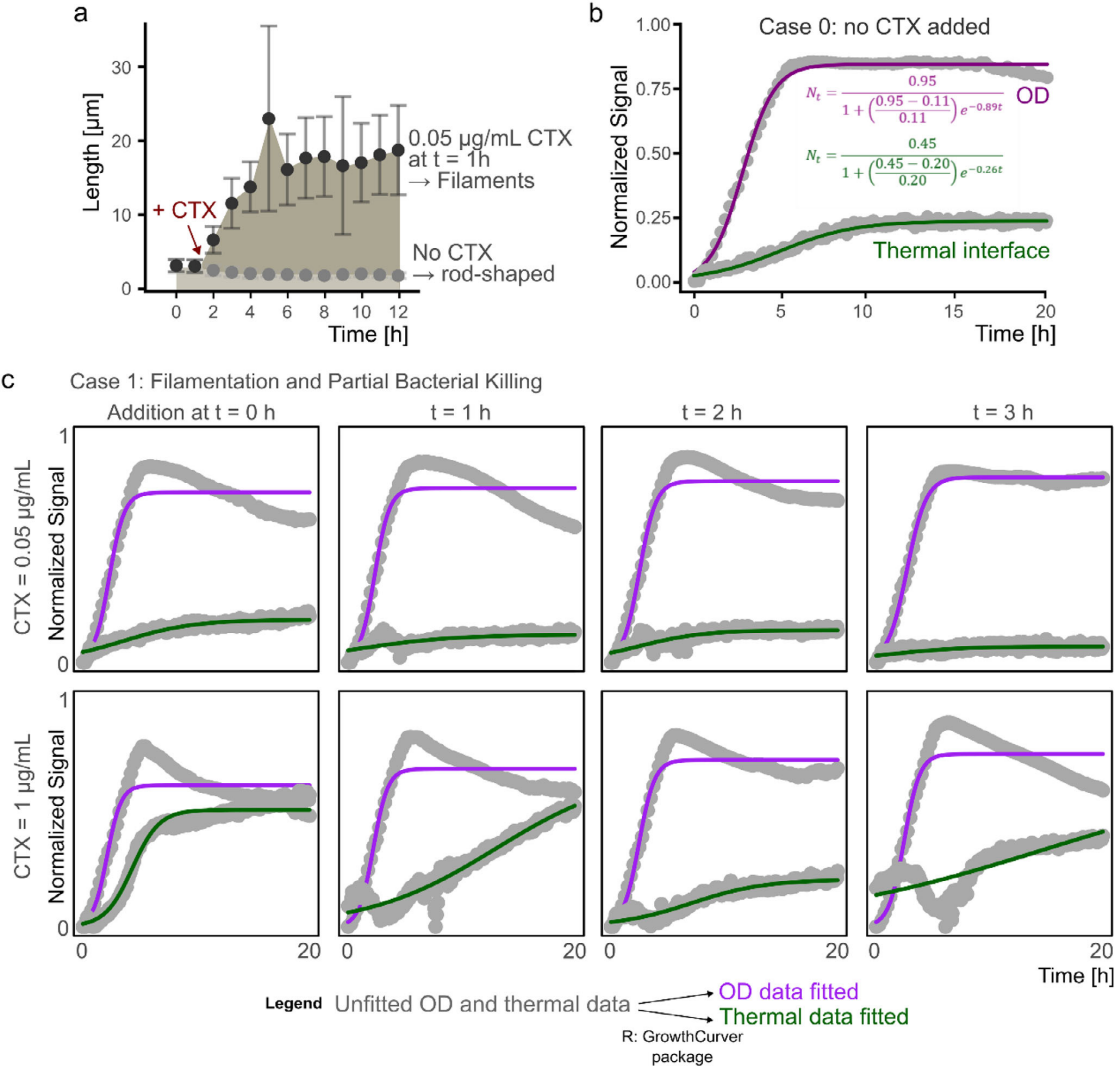
a) *E. coli* length assessment over 12 h using sequentially captured microscope images. In the presence of a sub‐MIC concentration of CTX, *E. coli* undergoes filamentation due to stress, while in the control group (no CTX), the cells remain rod‐shaped and small in size. The plot shows mean ± sd from n = 50 measurements. b) Thermal and OD data represented on a normalized and dynamic range transformed scale for case 0 (no CTX addition). c) Growth curve fitting for all experiments with sub‐MIC CTX addition, showing the kinetic growth patterns and the effects of antibiotic stress on *E. coli*.

In contrast, case 1 reflects bacterial growth after adding the antibiotics in the sub‐MIC concentrations (including 0.05 µg mL^−1^ and 1 µg mL^−1^) at the time points (t = 0, 1, 2, and 3 h) (see Figure 4c). Growth kinetics overall align with case 0 (no CTX addition) and reveal a similar trajectory, exponential and stationary phase, but with significant deviations in signal magnitude (see **Figure** **3**a i). Despite antibiotic pressure, continuous OD readings confirmed ongoing proliferation (see Figure 3a ii), although at reduced levels compared to case 0. However, the thermal response varied substantially depending on the degree of filamentation and partial bacterial killing. These morphological changes were confirmed by optical microscopy after 20 h of incubation and shown exemplarily for the condition of 0.05 µg mL^−1^ CTX at t = 1 h (see Figure 3a iii). Interestingly, the thermal signal did not always scale with OD. In some sub‐MIC conditions, the thermal signal exceeded that of the control, e.g., 1 µg mL^−1^ at t = 0 and 1 h, while in others it was lower (compare **Figure** **4**b,c). This discrepancy stems from morphological adaptations, particularly the formation of filamentous bacterial networks near the thermal interface, which OD does not capture with sufficient sensitivity. The extent of filamentation correlated with thermal signal magnitude: short, mildly filamented cells yielded lower thermal responses, while extensive filamentation produced stronger signals (Figure S5, Supporting Information). This effect highlights the responsiveness of the thermal interface to local structural and spatial changes in the proximity of the sensing interface, rather than just cell concentration. Thermal measurements in case 1 also showed slower apparent kinetics compared to OD, consistent with the interface's small sensing depth. Data normalization to a 0–1 scale allowed direct comparison of signal profiles between modalities and revealed that while OD reliably tracks total cell mass, the thermal signal provides complementary insights into cell morphology and assembly close to the interface. This becomes especially important under stress conditions like sub‐lethal antibiotic exposure, where filamentation and network formation near surfaces become dominant phenotypes.

In summary, while case 0 confirms expected bacterial kinetics, case 1 shows the added value of thermal sensing in conditions where the interface dominates physical changes, which can be seen in conditions when 1 µg mL^−1^ of CTX is added at t = 0 and 1 h. It captures interface‐dominated morphological changes that are not captured by OD measurements, proving the benefit of combining both sensing modalities for a more complete view of microbial growth dynamics under antibiotic stress. This observation on the emphasis of interface‐dominated rather than bulk‐dominated processes becomes even more pronounced in cases 3 and 4, which will be explained in detail in later sections.

### 3.3 Case 2 (CTX >> MIC): Complete Bacterial Lysis and Concordant Thermal‐Optical Response

Case 2 represents scenarios where *E. coli* cultures are exposed to CTX concentrations far above the MIC level, resulting in full bacterial lysis. Under these conditions, the thermal signal exhibits a characteristic dip followed by stabilization at baseline levels (from 2 h until 4.5 h). This indicates a cessation of growth and a drastic reduction in viable biomass near the sensing interface (see Figure 3b i). The thermal signal drop is attributed to the rapid decline in cellular content and, thus, *e* in the sensing zone due to cell lysis.

This response was observed across multiple conditions, including 250 and 500 µg mL^−1^ CTX administered at t = 0 and 1 h. In all cases, the thermal signal remained low after the initial dip, showing no recovery, and hence further supporting the interpretation of complete bacterial clearance. The OD data mirrored these findings, confirming lysis across the entire bulk volume (see Figure 3b ii). Analysis by optical microscopy after 20 h of cultivation revealed only cellular debris, with no viable or filamentous bacteria remaining (see Figure 3b iii).

Unlike case 1 (bulk‐dominated events), where the thermal interface revealed signal features not captured by OD due to local effects, case 2 demonstrates a strong convergence between the two sensing modalities. Both techniques detect the same biological event and reflect it with similar trends, emphasizing their complementary validity. However, this finding also highlights that under conditions of spatially uniform cell death (without distinct behaviors near the thermal interface), thermal sensing does not provide additional discriminatory power over conventional OD. The combination of both techniques could be leveraged as a powerful screening tool to identify new antibiotics.

### 3.4 Case 3 (CTX > MIC): Insights into Filamentation Dynamics Before Complete Lysis – The “Hill” Effect

**Figure 5:**
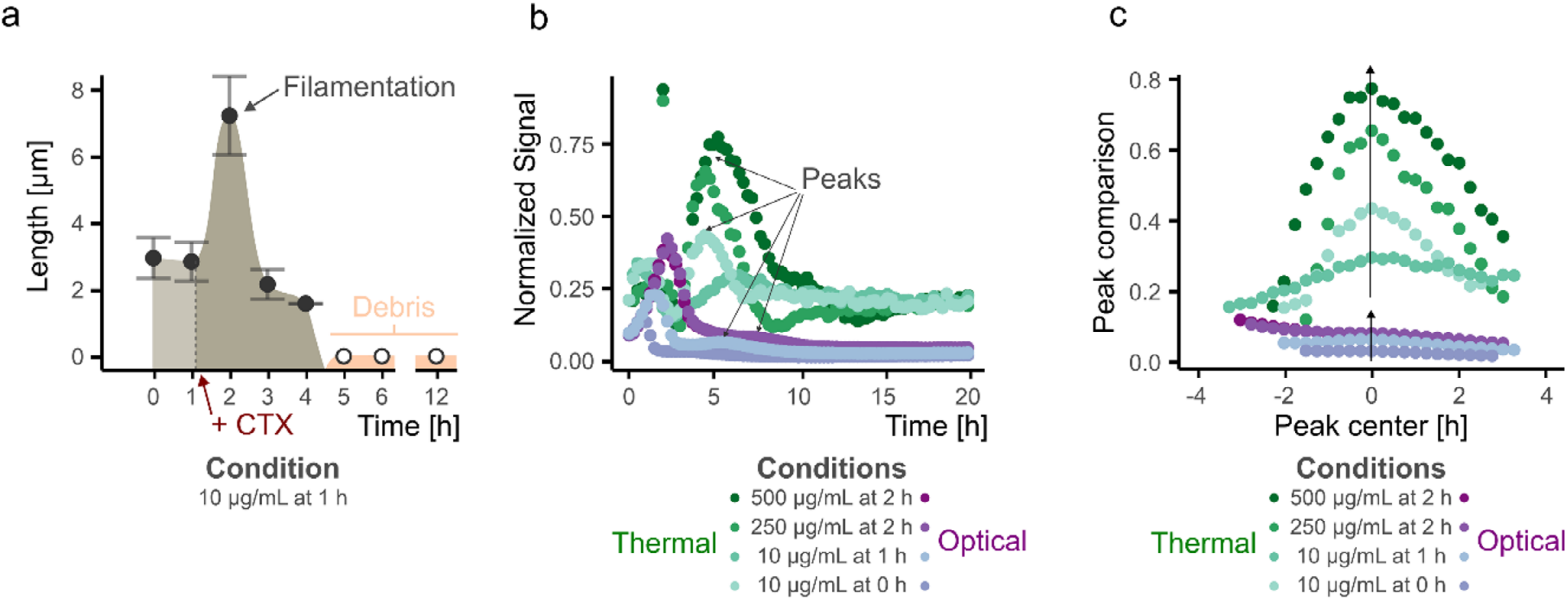
a) Morphological changes of the *E. coli* culture over time under the condition of 10 µg mL^−1^ CTX added after 1 h of incubation. Filamentation and cell elongation are observed as a response to antibiotic stress. The plot shows mean ± sd from n = 50 measurements. b) All conditions falling under Case 3 are plotted on top of each other. The distinct “hill effect” peaks are more pronounced in the thermal data, highlighting the filamentation response. Data is presented on a normalized and dynamic range‐transformed scale. c) Peak extraction and centering for better comparison of peak intensity differences. Higher CTX/bacteria ratios lead to larger peak intensities, illustrating the influence of antibiotic concentration on the thermal response.

Unlike case 2 (complete bacterial lysis), case 3 presents a distinct dip‐and‐hill profile in the thermal signal, revealing a dynamic two‐phase bacterial response to CTX stress. The first part of the two‐phase response is due to the antibiotic exposure that causes a signal drop similar to case 2 (complete bacterial lysis), indicating initial bacterial lysis. However, instead of stabilizing at baseline, the thermal and OD signals recover and peak before eventually returning to the lower levels, which defines the second part of the response (see Figure 3c i–iii). This “hill” is attributed to a temporary regrowth phase characterized by stress‐induced filamentation, where surviving *E. coli* elongate as a defense mechanism before succumbing to sustained antibiotic pressure (see **Figure** **5**a). This pattern is observed under intermediate CTX exposure conditions, such as 10 µg mL^−1^ at t = 1 h, and 250–500 µg mL^−1^ at t = 2 h.

Microscopy images taken each hour sequentially confirm the presence of long filamentous bacteria alongside increasing cell debris, supporting the interpretation of partial recovery followed by clearance (see Figure 5a). The OD data mirrors this general trend, with an initial drop followed by a modest rise; however, the thermal signal more strongly accentuates the peak on a normalized and dynamic range transformed scale (see Figure 5b), reflecting localized accumulation of filamentous cells at the sensor surface. This contrast stems from the different measurement principles (interface‐dominated vs bulk‐dominated).

Rescaling of the dynamic range to a scale between 0 and 1, region of interest extraction (i.e., peak extraction), and peak centering further reveal this divergence (see Figure 5c). Relevant peaks or plateaus were identified after CTX addition, as the earlier maxima reflect uninhibited exponential growth, and, thus, do not represent the response to the morphological adaptation. While OD typically shows a weak peak or plateau, thermal signals produce more pronounced maxima, suggesting an increased signal contribution from sedimented filamentous cells. This behavior is further modulated by the ratio of antibiotic concentration to bacterial density (CTX/bacteria), which was used as a parametric factor to compare conditions across time points and concentrations (for calculation details, Table S3, Supporting Information). In general, higher CTX/bacteria ratios correspond to stronger filamentation and larger signal peaks. This could be because the more viable bacteria are available upon CTX exposure, the greater their ability is to cushion the stimulus‐induced stress.

These observations highlight the strength of thermal sensing in interface‐dominated biological processes. The morphological adaptation and partial regrowth were detected with a higher response signal, reinforcing that the signal trend cannot be fully captured or explained by bulk techniques alone. This local responsiveness is biologically relevant in contexts where surface‐specific responses are critical, such as the early stages of plaque or biofilm formation on medical implants. It enables the detection of subtle, spatially confined bacterial behaviors that may precede more severe infections. This becomes increasingly important in more complex response scenarios, as demonstrated here for case 3 and further underscored in the upcoming case 4.

### 3.5 Case 4 (CTX > MIC, but CTX Added at Later Time Points): Persister Cell Formation and Interface‐Specific Signal Shifts

**Figure 6:**
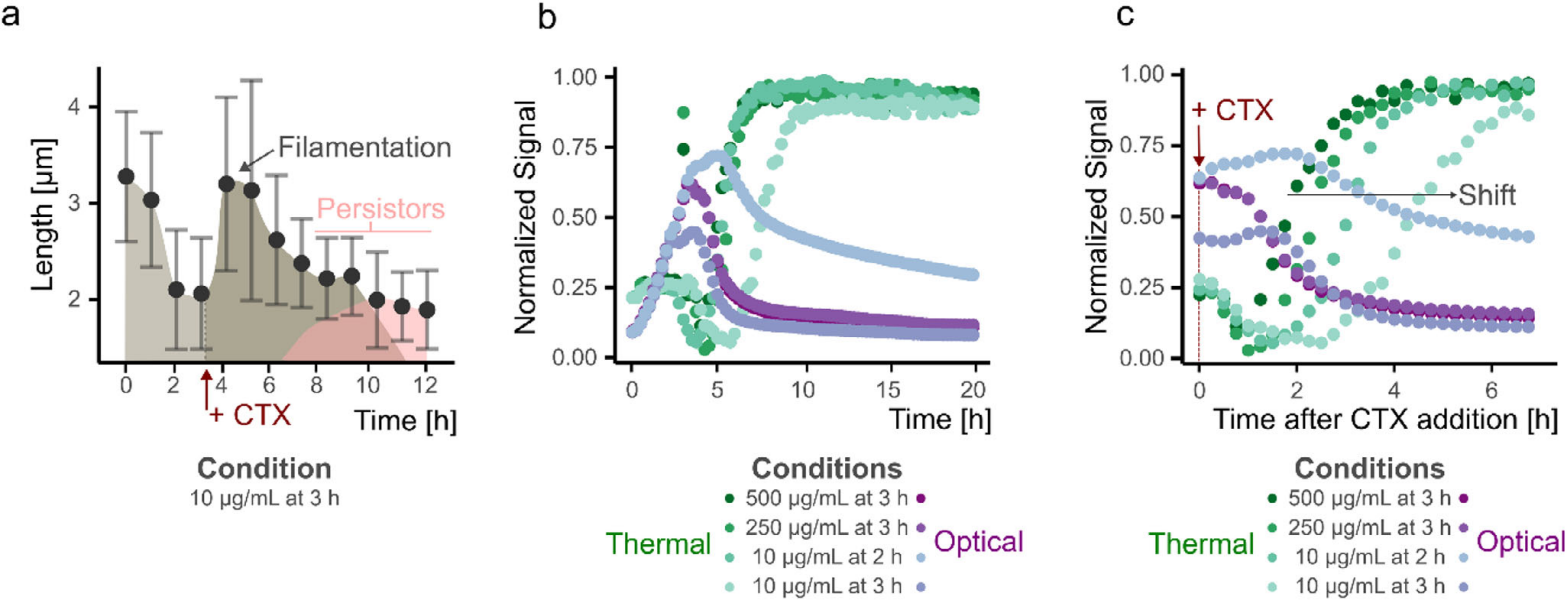
a) Morphological changes of the *E. coli* culture over time under the condition of 10 µg mL^−1^ CTX added after 3 h of incubation. Filamentation and the transition to normal‐sized bacteria are observed as the culture responds to antibiotic stress. The plot shows mean ± sd from n = 50 measurements. b) All conditions classified as Case 4 are plotted on top of each other. The difference in data trends between thermal and optical data is observed. In the thermal data, a distinct jump and stabilization to a higher baseline level can be seen, indicating persister cell formation and survival. Data is presented on a normalized and dynamic range‐transformed scale. c) Signal jump extraction from the time point of CTX addition until stabilization of the signals. The shift in kinetics is observed, with faster kinetics for higher CTX/bacteria ratio conditions, demonstrating the influence of antibiotic concentration on the rate of persister cell formation.

Case 4 reveals a complex bacterial response characterized by an initial dip in thermal signal (from 3.5 to 7 h), followed by a sharp “jump” and eventual stabilization at an elevated level (from 7 to 9 h) (see Figure 3d i). This unique thermal profile contrasts with prior cases and suggests a multi‐phase survival strategy involving both morphological adaptation and metabolic persistence. The observed signal dip reflects initial bacterial lysis due to high‐dose CTX exposure, similar to cases 2 (CTX >> MIC) and 3 (CTX > MIC). Shortly after antibiotic administration, mild filamentation is observed as a rapid survival strategy, which can be observed minutes to a few cell cycles after CTX exposure. Bacteria are observed to elongate up to three times their normal length (see **Figure** **6**a). These filamentous forms, however, are still susceptible to CTX and gradually lyse under stress.

The thermal signal then exhibits a pronounced increase, a jump not mirrored in the OD data (see Figure 3d ii). Microscopy confirms that this jump coincides with the emergence of persister cells: dormant, antibiotic‐tolerant *E. coli* that survive the stress and gradually revert to a non‐filamentous, single‐cell morphology. This survival strategy is known to be slower, since it requires stochastic entry or stress‐induced signaling cascades, which becomes evident after hours of antibiotic exposure. 7–8 h post‐antibiotic treatment, bacterial length decreases and stabilizes ≈2 µm, consistent with normal *E. coli* dimensions (see Figure 3a iii) and Figure 6a). These surviving cells repopulate the interface region, leading to the elevated thermal signal plateau.

Importantly, the signal shift is not observed in the OD measurements, which remain relatively flat or show only minimal changes (see Figure 6b). This reinforces the unique strength of the thermal interface in detecting localized and subtle changes in bacterial morphology and behavior. The microscopy data and follow‐up recovery experiments confirm that these bacteria retain the ability to regrow under favorable conditions, validating the presence of true persisters (Note S4, Supporting Information).

The progression and magnitude of these events are influenced by the CTX/bacteria ratio (Table S3, Supporting Information). Higher ratios induce quicker transitions and more pronounced signal shifts, whereas lower ratios delay the onset of signal change (see Figure 6c). Still, it is the thermal signal that most clearly reflects these transitions, due to the sedimentation of elongated or dense bacterial subpopulations at the sensor interface, filaments early on, and later, clusters of persister cells (see Figure 6c).

This case underscores the responsiveness of the thermal sensing in interface‐dominated scenarios. While OD provides valuable bulk information, it fails to capture the detailed temporal and morphological dynamics occurring near the surface. The ability of the thermal interface to resolve interface‐specific phenomena like filamentation, local lysis, and bacterial persistence highlights its strength in detecting subtle stress responses. This is biologically relevant in the context of antibiotic resistance, where persister cells can evade treatment and contribute to recurrent infections, an ongoing challenge in clinical environments such as hospitals. In the future, this approach could aid in the rapid identification of stress‐adapted subpopulations, helping to evaluate antibiotic efficacy and inform more effective treatment strategies.

## 4 Conclusion

We presented a reusable, flexible, and noninvasive thermal interface that integrates seamlessly into standard microplates and combines localized heating with real‐time effusivity‐based sensing. This dual‐function system allows for precise, well‐specific temperature control and dynamic monitoring of sample properties, without direct contact between electrodes and the liquid. Using bacterial proliferation as a representative use case, we validated the platform's performance by monitoring *E. coli* growth under various conditions, including the spiking of the bacteriolytic CTX in a wide concentration range and the administration at different time points. For the first time, a dual‐function thermal interface has been used to deeply investigate morphological and metabolic responses of bacteria to CTX stimulus. The results demonstrate that the thermal interface captures general growth trends with a specific emphasis on interface‐dominant processes. These interface‐dominant dynamics, like filamentation or bacterial persistence, cannot be resolved with sufficient sensitivity in OD measurements. The thermal technique benefits from the natural tendency of *E. coli* to sediment under static culture conditions, which enhances interface responsiveness. However, because the sensing depth extends well beyond the adherent cell layer (≈500 µm), the method remains applicable to non‐adherent bacteria, albeit with some potential loss in response signal. Analysis of the underlying mechanisms of these adaptations can be relevant for understanding antibiotic resistance, chronic infections, and treatment failure. Quantitative growth parameters and MIC can be derived using standard statistical tools and established libraries (shown for the R package: growthcurver). We introduced a new data analysis regime, giving mTPS data more robustness and making it less prone to systematic errors. The current platform already offers key features, such as operation without electrical contact with the liquid, reusability after sterilization, and spatially localized sensing within a microplate format. These findings open the door for broader applications. Given that microbiological assays are still largely dominated by agar plate–based techniques, the presented method offers a path toward automating such interface‐dominated assays, which are currently performed manually or under high‐resolution microscopy in clinical antimicrobial resistance testing. In contrast to imaging‐based methods that require bulky, high‐magnification optics and frequent dilution to avoid surface overgrowth, the thermal interface provides a compact, scalable, and label‐free alternative for monitoring bacterial dynamics at relevant interfaces. Beyond microbiology, this approach holds promise for real‐time analysis of surface‐dominated processes such as polymerization, biofilm formation, or cell adhesion. The system's energy efficiency, compatibility with automation, and ease of integration into compact, electronic assay platforms make it a compelling candidate for next‐generation microTAS, lab‐on‐a‐chip systems, or electronic microplates. By introducing a new dual‐function thermal modality into standard microplates, this work paves the way for multi‐parameter and real‐time assays that bridge physical, chemical, and biological domains in a single, modular platform.

## 5 Experimental Section

### Fabrication of Sensors

The sensors were fabricated on a flexible, single‐sided copper‐clad polyimide laminate (AC182500E, CCI Eurolam GmbH) using a process based on photolithography and chemical etching. The copper‐clad polyimide laminate sheets were cut into 5 cm × 5 cm substrates, followed by an IPA bath for 2 min and dry baking at 120 °C for 3 min. The substrates were then treated with O_2_ plasma (10 Pa, 15 sccm) at 100 W for 30 s. A TI Prime adhesion promoter was spin‐coated at 3000 rpm for 30 s, followed by baking at 120 °C for 2 min. AZ5214e photoresist was then spin‐coated at 4000 rpm for 30 s and baked at 100 °C for 90 s. The double‐spiral‐shaped sensors were patterned using direct laser writing with the 40 mm writer head, laser power of 185 mW, and 100% intensity (DWL66+, Heidelberg Instruments). After patterning, the photoresist was reverse‐baked at 120 °C for 2 min. The substrates were then flood‐exposed using a UV exposure box (UV Exposure Box 1, Gie‐Tec GmbH). To reveal the structures, the substrates were developed in AZ351B:DI water (1:4) for 45 s, followed by rinsing with DI water and drying. The uncovered copper was chemically etched using a 40% (w/v) FeCl_3_ solution. The etching process involved immersing the substrates in the etching solution for 150 s, followed by two rinses in DI water. This cycle was repeated until the sensing structure was fully etched and had a clean finish (8–10 cycles, total etching time of 20–25 min). Finally, the sensors were cleaned in a series of baths: 2 min in acetone, 2 min in IPA, and 2 min in DI water. Microscopy images and height profiling can be seen in Figure 1a and Figure S4 (Supporting Information).

### Fabrication of an In‐House Modified Microplate

No‐bottom 24‐well microplates (Greiner Bio‐One 24‐well No‐Bottom Microplates) were purchased and modified in‐house for reusable, leak‐proof integration of the flexible sensors; the modification process is schematically shown in Figure S3 (Supporting Information). A 10:1 PDMS‐to‐curing agent mixture was poured into a mold to a 2 mm filling level. The microplate was then placed into the mold and gently pressed to the bottom. The mixture was cured at RT for 48 h. After curing, the microplate was cut along its dimensions with a scalpel and detached from the mold. The wells were also opened using a scalpel. Before the attachment of sensors, the PDMS was wiped with 70% vol. ethanol and dedusted with residue‐free adhesive tape. Sealing tests were performed to assess the attachment of polyimide‐based flexible sensors (Figure S3, Supporting Information).

### M9 Cell Culture Medium Preparation

M9 medium for the experiments was prepared in‐house in batches of 400 mL. The medium was stored at +4 °C. To prepare the M9 salt solution, 80 mL of 5 × M9 minimal salt solution (Sigma–Aldrich) was added to 320 mL of Milli‐Q water to obtain 1 × M9 minimal salt solution. The salt solution was sterilized by autoclaving at 121 °C for 20 min.

To prepare the M9 compound media, 40 mL of the stock M9 minimal salt solution was supplemented with 3.2 g of glucose (Sigma–Aldrich), 400 mg of MgSO_4_∙7H_2_O (Sigma–Aldrich), and 200 mg of casein hydrolysate (Sigma–Aldrich). After dissolving and mixing, the additives were sterilized by filtration using a sterile syringe and filter (VWR, 0.2 µm cellulose acetate membrane). Finally, the autoclaved M9 salt solution and the sterile‐filtered additives were combined to obtain the final M9 media.

### *E. coli* YFP Pre‐Culturing

*E. coli* YFP (MG1655 galK::SYFP2‐FRT) was used for all experiments.^[^ ^35^ ^]^ For the initial long‐term stock, a single colony was transferred from an agar petri dish into a sterilized flask containing M9 media and incubated until the culture reached the mid‐exponential phase. The stock was then mixed with 70% glycerol and stored at −80 °C. During the experimental phase, the *E. coli* cultures were maintained as short‐term stock. A small amount of frozen stock was scraped off with a sterile inoculation loop and transferred to a sterile Erlenmeyer flask containing 20 mL of M9 media. This culture was incubated overnight (T = 37 °C; 170 rpm shaking) for recovery. For the experiments, 19 mL of fresh M9 media was mixed with 1 mL of the short‐term stock and incubated (T = 37 °C; 170 rpm shaking) until the culture reached mid‐exponential phase (typically 160–200 min, OD_600_ = 0.4–0.8 A). The short‐term liquid batch was used to prepare the starting concentration for each experiment and was temporarily stored at 4 °C until the next pre‐culture was needed.

### *E. coli* Growth Monitoring and Antibiotic Testing

The goal of this experimental series is to demonstrate that the sensor can simultaneously control the temperature to maintain optimal bacterial growth conditions (37 °C) while dynamically monitoring bacterial proliferation.

Bacteria were seeded in microplate wells with an initial OD_600_ = 0.1 A (5.1 × 10^7^ cells mL^−1^) with a total volume of 2 mL of fresh M9 media. To ensure reproducible starting conditions, each experiment was performed using a freshly prepared *E. coli* pre‐culture, as described previously. The measurement parameters were set as follows: T_Setpoint_ = 37 °C, P_Pulse_ = 100 mW, t_Pulse_ = 2 s, t_Temperature control_ = 178 s, and a sampling frequency of 50 Hz (B2902A Precision Source/Measure Unit, Keysight). Each condition was measured in triplicate to verify reproducibility. Additionally, source measurement unit (SMU) channels and thermal sensors were randomly assigned to minimize statistical errors caused by the measurement setup and sensor variability (Table S2, Supporting Information).

To assess the effect of antibiotics, bacterial cultures were exposed to CTX at different concentrations (0.05, 1, 10, 250, and 500 µg mL^−1^) at various time points: 0 h (start), 1, 2, and 3 h after the onset of growth. All conditions and repetitions are listed in Table S2 (Supporting Information).

### Heating Element Assessment

Temperature control was evaluated using an infrared camera (U5855A TrueIR Thermal Imager, Keysight). Images were captured in a dry state to assess the PID control. The temperature of the mTPS element was recorded from a distance of 10 cm through the polyimide substrate. Images were taken 1 min after initiating temperature control. The experimental setup is shown in Figure S2 (Supporting Information). The accuracy of the measured temperature compared to the setpoint was calculated.

### Statistics and Reproducibility

Statistical analyses were performed in RStudio 2024.12.0+467 and Microsoft Excel for Microsoft 365 MSO (Version 2508 Build 16.0.19127.20192). All bacterial experiments were conducted with three independent technical replicates (n = 3). Details on data representation and sample size are included in figure legends. The experiments were randomized, Table S2 (Supporting Information). Growth kinetics for case 0 and case 1 were calculated using the growthcurver R package, Table S1 (Supporting Information). Additional relative absorbance unit (RAU) and lower limit of detection (LOD) calculations were performed following the analysis of Chen et al. ^[^ ^36^ ^]^ The results can be seen in Table S3 (Supporting Information).

### Cell Density Calculation

The cell density for the seeding concentration of *E. coli* for the growth experiments was measured by Biophotometer (Eppendorf) and calculated with the following formula (M9 compound medium). Before measuring the cell density, the pre‐culture was diluted into the measuring range of 0 ≤ OD_600_ ≤ 1. The result is obtained by multiplying the dilution factor.

(1) $$Cell\;density=5.1\times 10^{8}\times OD_{600}\left(0\leq OD_{600}\leq 1\right)\left[\frac{Cells}{mL}\right]$$

The cell density calibration for the microplate reader was obtained by the preparation and measurement of a dilution series in the linear range of the plate reader. The signal was further calibrated with the cell density measured by OD_600_.

(2) $$Cell\;density=7.5\times 10^{8}\cdot OD_{Plate}-1.7\times 10^{7}\left[\frac{Cells}{mL}\right]$$

## Conflict of Interest

The authors declare no conflict of interest.

## Supporting information



Supporting Information

## Data Availability

The data and code that support the findings of this study are available from the corresponding author upon reasonable request.
