# Supplementary material for: Dual‐function, Reusable, and Flexible Thermal Interface for Kinetic Monitoring of In Vitro Bioassays
Source: Small Methods. 2025 Dec 18;10(3):e01243. doi: 10.1002/smtd.202501243 (PMC12893297; doi:10.1002/smtd.202501243)
Supplement: Supplementary file 1 — Supporting Information [file SMTD-10-e01243-s001.docx]

Supplementary File

Dual-function, Reusable, and Flexible Thermal Interface for Kinetic Monitoring of *In Vitro* Bioassays

Daniel Nieder^1^, Isli Cela^1^, Željko Janićijević^1^, Xinne Zhao^1,2^, and Larysa Baraban*^1,2^

^1^ Institute of Radiopharmaceutical Cancer Research, Helmholtz-Zentrum Dresden-Rossendorf (HZDR), Bautzner Landstraße 400, 01328 Dresden, Germany

^2^ Else Kröner Fresenius Center for Digital Health, Faculty of Medicine Carl Gustav Carus, Technische Universität Dresden, Dresden, Germany

***Corresponding authors: [l.baraban@hzdr.de](mailto:l.baraban@hzdr.de)

***SI Note 1.* Reusable and integratable thermal interfaces for commercial microplates**

Figure 1a illustrates the detailed design and integration of the in-house fabricated thermal interface into a commercially available, but modified, 24-well microplate. The flexible thermal interface is constructed on a 25 µm-thick polyimide substrate and features a double-spiral thermal element with a diameter of 4.7 mm. The element’s width, spacing, and height are 63.6 ± 4.9 µm, 63.6 ±4.6 µm, and 13 µm, respectively (see Figure S3). This design allows seamless integration into the modified microplate (see Figure 1d).

To ensure experimental reliability, leak tests were performed, confirming a secure seal throughout the entire assay with no cross-contamination between wells (Figure S3). The integration process is highly reproducible and achieved by modifying commercially available bottomless microplates with PDMS, which provides a tight seal while maintaining compatibility with standard laboratory workflows (see the modification protocol in Figure S3). All materials are fully sterilizable via ethanol treatment and autoclaving, ensuring reusability across multiple experiments.

The thermal interface demonstrated exceptional long-term stability and reusability. Throughout six months of continuous use, spanning over 50 experiments, a single microplate maintained full functionality without any degradation in performance. The dual functionality of the sensor is achieved through a non-contact approach, where only the polyimide top side of the interface is in direct contact with the sample (see Figure 1a). This eliminates concerns regarding fouling or corrosion.

Furthermore, only four thermal interfaces were required for the entire experimental series, operating within the same timeframe as the microplate itself. No loss of functionality was observed. This level of durability positions the thermal interface as a sustainable alternative to conventional single-use sensors, such as those employed in electrochemical impedance spectroscopy. By reducing waste accumulation and extending sensor lifespan, this approach contributes to more sustainable and cost-effective bioassay methodologies.

***SI Note 2.* Temperature control assessment**

The functionality of the heating element for temperature control is demonstrated in Figure 1h, i,j. For microheaters, a proportional-integral-derivative (PID) control feedback system is widely used due to its precise temperature regulation, ability to minimize overshooting, and fast response time. In this study, the PID weighting factors were optimized to achieve a rapid response while preventing temperature overshoot, with values set at k_p_ = 7, k_i_ = 0.5, and k_d_ =0.01. A schematic illustration of the PID control theory is provided in Figure S1. Heating performance was assessed using a thermal infrared camera, showing stable temperature values within **<60 s** after activation. The system was tested in the temperature range of 25–40 °C, covering all relevant ranges for most bioassay applications. Figure 1j displays thermal heat maps of the heating element, revealing a uniform heat distribution. Additional heat maps corresponding to each temperature step are provided in Figure S2.

The temperature control system demonstrated an average accuracy of **99.56 ± 0.37%.** These assessments were conducted in air; however, given that water has a higher heat capacity (c_p_(air) = 1 $\frac{J}{g K}$ and c_p_(water) = 4.2 $\frac{J}{g K}$), temperature stability is expected to be improved when liquid samples are present in the wells.

***SI Note 3.* Robust Data Analysis**

Traditionally, data analysis in mTPS measurements relies on linear regression applied to a manually selected interval of the heating curves. However, this approach is susceptible to random errors, as the choice of interval boundaries and interval width significantly impact the results. The effect of interval selection is demonstrated in Figure S6, where selecting an interval at the end of the heating curve reveals a kinetic change in thermal properties, whereas an interval closer to the center of the heating curve loses all distinguishing features. To mitigate these inconsistencies and establish a more robust, reproducible analysis method, we propose an approach that eliminates human-dependent interval selection. Instead of relying on predefined sections of the heating curve, we fit the entire heating curve using a second-order polynomial function of *y = ax^2^+bx+c*, where the coefficient *a* consistently shows reproducibility and statistical significance, making it a reliable parameter for further data processing. Coefficient *b* has also been tested as a measure to observe these trends, but it expresses a lower signal-to-noise ratio. This method ensures that the analysis captures the full thermal response of the system, improving sensitivity to dynamic changes in thermal properties over time. By avoiding manual selection of analysis windows, this approach enhances data consistency, reduces human error, and provides a more objective assessment of thermal behavior in complex biological systems. The kinetics plots are presented as the percentage change in coefficient *a*, calculated relative to the median coefficient of an initial baseline interval at the beginning of the experiment. This normalization step compensates for differences in the initial resistance (*R*_init_) between sensors. Because the assay starts at room temperature (RT) and the system is then heated to the setpoint (37 °C), a transient phase occurs before thermal equilibrium is reached (<15 minutes). During this period, the rapid temperature increase leads to pronounced changes in sensor resistance, which can appear as negative percentage values when referenced to the reference point (median value of the first 10 datapoints). To prevent misleading interpretation of this transient behavior, all negative percentage values before the reference point are set to zero during data processing. These kinetics plots are then plotted as the percentage change in coefficient *a* relative to the coefficients measured in an interval at the beginning of the experiments. This last step of presenting the percentage change eliminated the influence of the difference in initial resistance *R*_init_ between sensors.

***SI Note 4*. Validation of Persister Cell Formation**

The presence of persister cells was validated through a regrowth experiment under favorable culture conditions following the initial 20-hour treatment period. Bacterial cultures exposed to 10 µg/mL CTX added at 2 h and 3 h were selected as representative conditions for case 4. After 20 hours of incubation, the cultures were collected and washed using a two-step protocol to remove residual CTX from the medium. This involved centrifugation (6700 rpm, 5 min), followed by two rounds of resuspension in PBS, and a final resuspension in fresh M9 medium. The washed cultures were then incubated overnight at 37 °C in a 24-well microplate. After incubation, OD₆₀₀ values of 0.614 (CTX added at 2 h) and 0.604 (CTX added at 3 h) were measured, indicating active bacterial growth. These results confirm the presence of viable persister cells capable of resuming growth once antibiotic stress is removed, thereby validating the occurrence of true persister formation under these conditions.


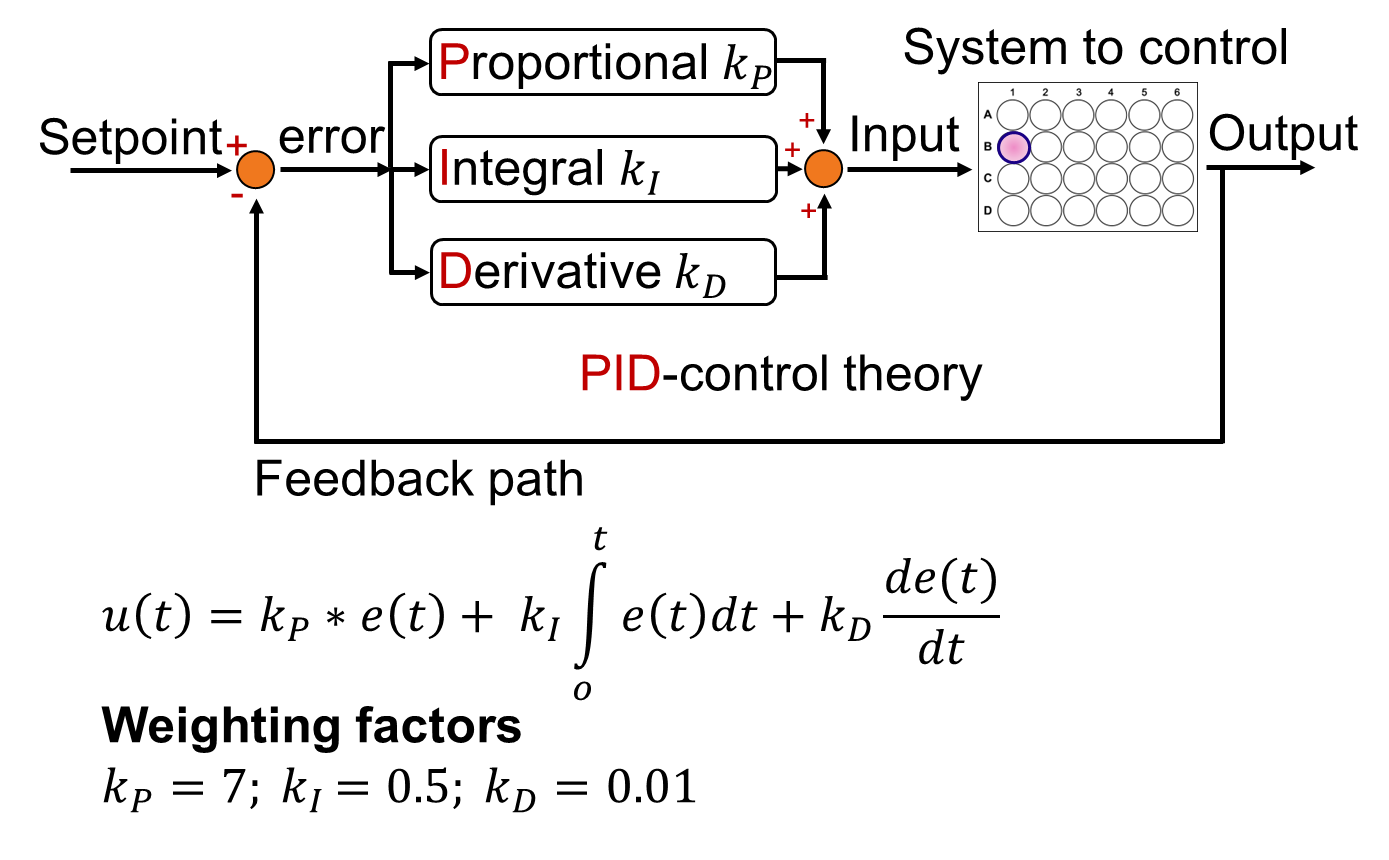


***Control theory***


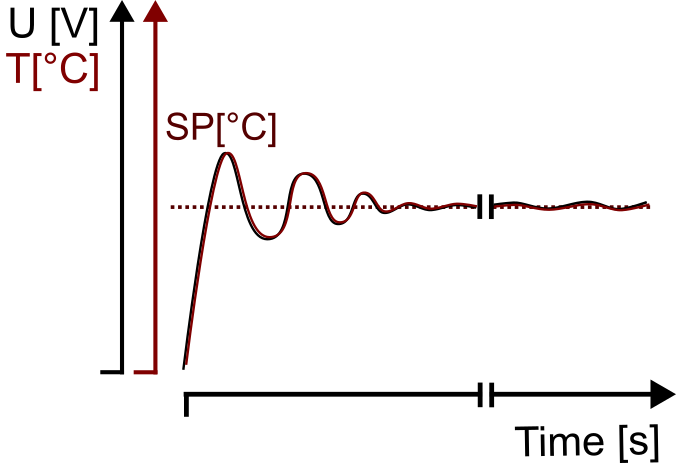


**Figure S1.** Control scheme of a proportional-integral-derivative (PID) controller used for thermal regulation of the sensor interface. The PID controller continuously adjusts the heating input based on the difference between the setpoint and the actual temperature (error signal). The proportional term provides an immediate response to the current error, the integral term corrects for accumulated past errors, and the derivative term anticipates future errors by reacting to the rate of temperature change. Together, these components ensure fast and stable temperature convergence with minimal overshoot. The weighting factors for each term (P, I, and D) were experimentally tuned to achieve optimal performance specific to the thermal mass and response dynamics of the microplate-based heating system.


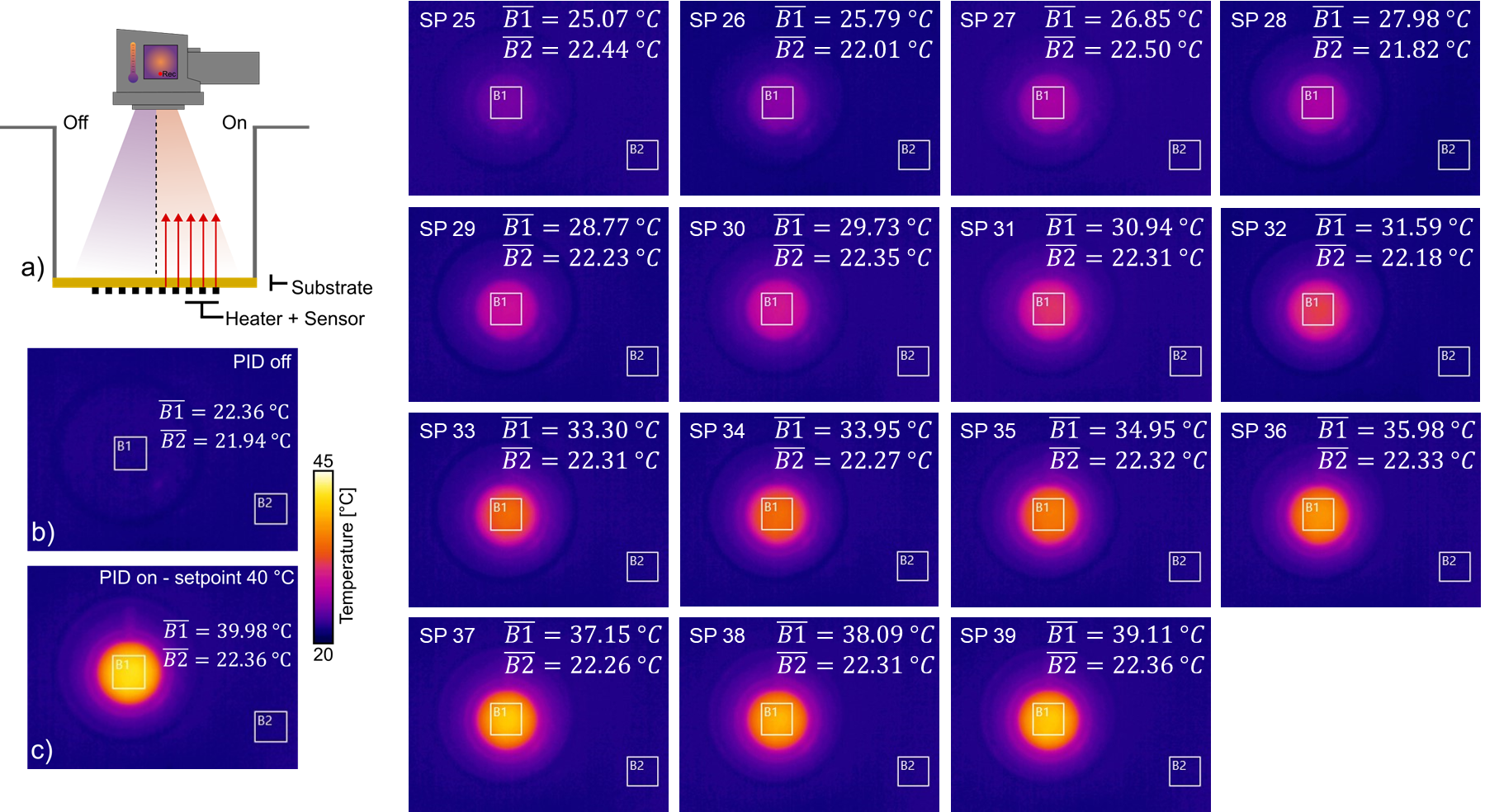


**Figure S2**. a) Experimental setup used to evaluate temperature control and stability of the thermal interface system. b) Thermal map recorded without active temperature control (PID off), illustrating spatial temperature variability. c) Thermal map with PID temperature control activated, maintaining the setpoint of 40 °C. Additional thermal maps on the right depict the controlled temperature distribution across the relevant operational range (25–39 °C). Temperature accuracy was determined using the average temperature within region **B1** (target measurement zone), while **B2** served as a reference for background temperature.


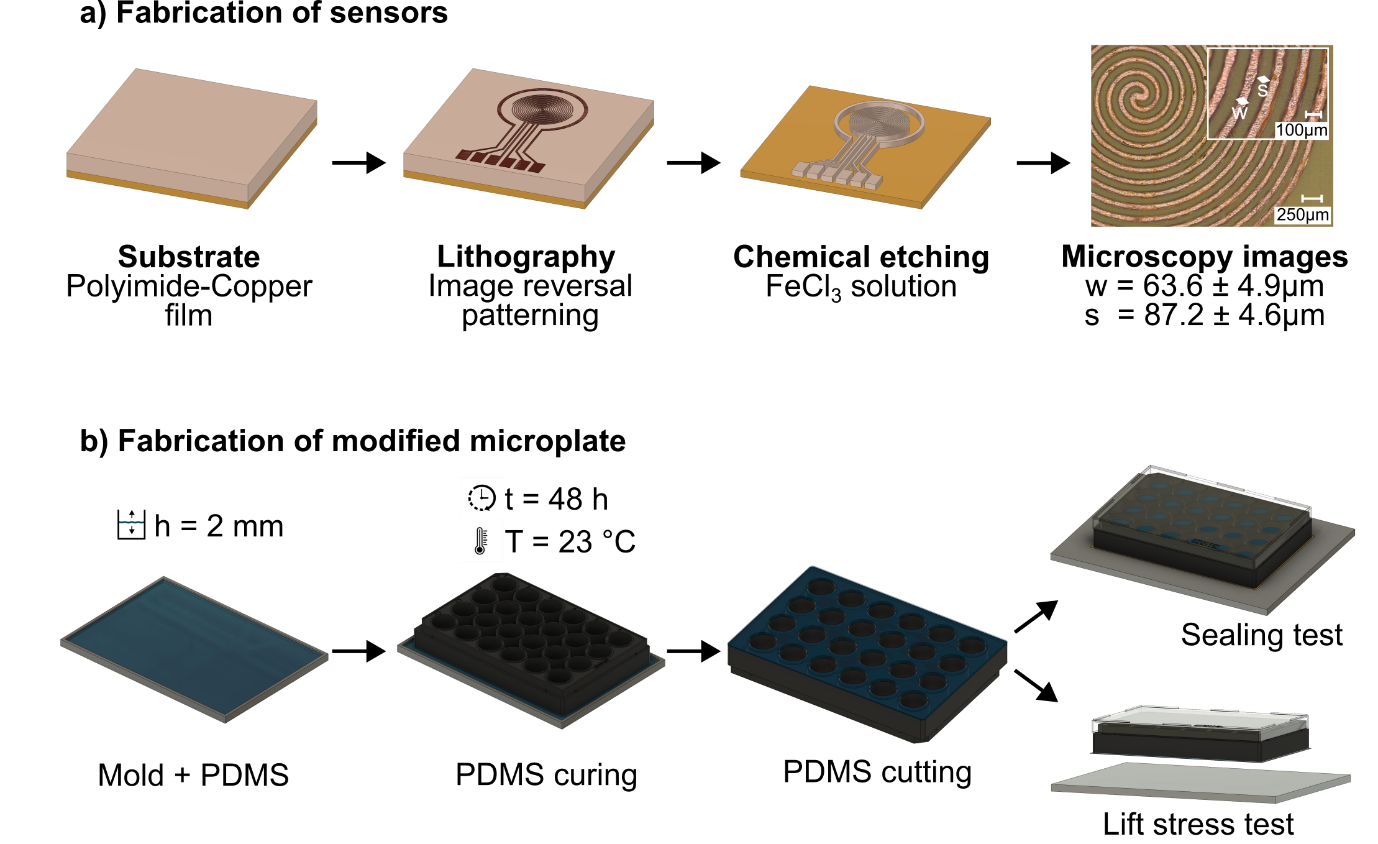


**Figure S3.** **a)** Schematic illustration of the fabrication process of the thermal interface. The process involves photolithographic patterning followed by a chemical etching step to define the sensor geometry. A representative microscope image shows the resulting average track width and spacing, confirming consistent and reproducible fabrication quality.
**b)** Flow diagram of the modification steps for the in-house adapted 24-well microplate. A sealing test and mechanical stress test were conducted to validate leak-proof integration of the thermal interface and to demonstrate the microplate’s reusability without compromising its structural integrity or sensing functionality.

#
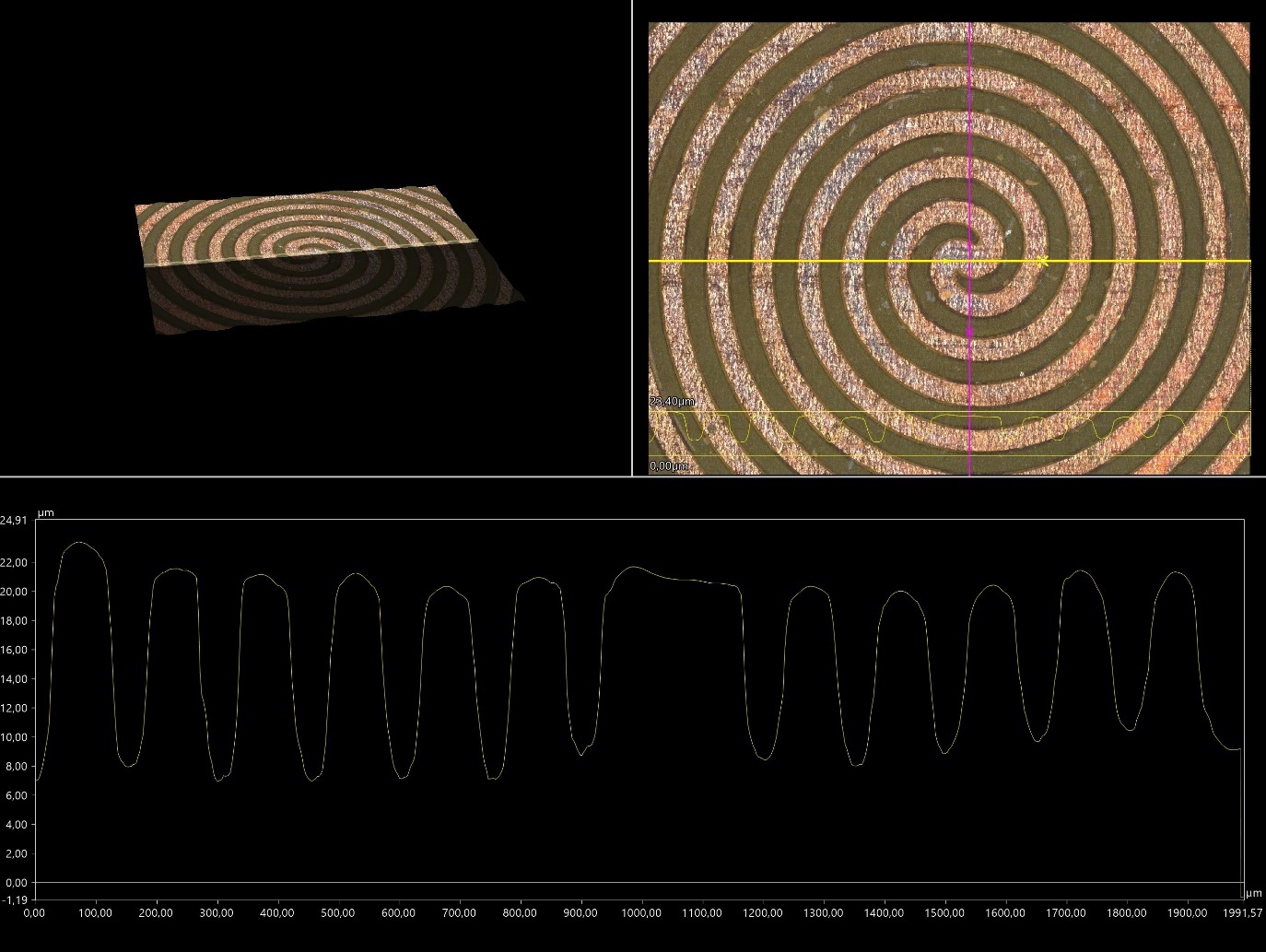


**Figure S4.** Microscope image and height profile analysis of the thermal interface acquired using the Keyence VHX-7000 digital microscope. The cross-sectional height profiling confirms a clean and well-defined etching result, with sharp boundaries and uniform depth across the interface. This ensures consistent thermal contact and reliable measurement conditions during sensing experiments.

#
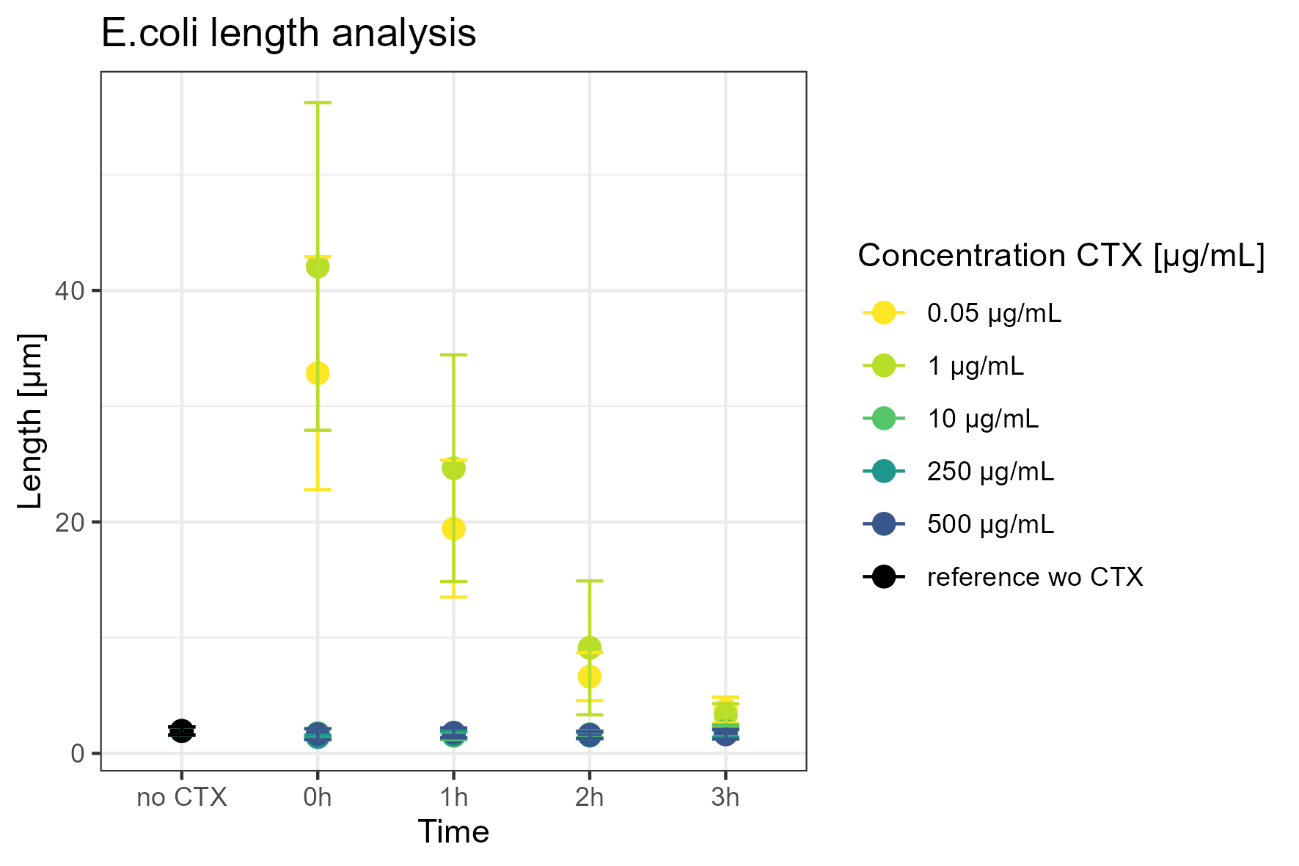


**Figure S5.** Average length of E. coli filaments after 20 h of incubation under different conditions. For sub-MIC concentrations of CTX, the length decreases with the delay of the addition time point. The plot shows mean ± sd from n > 50 measurements.


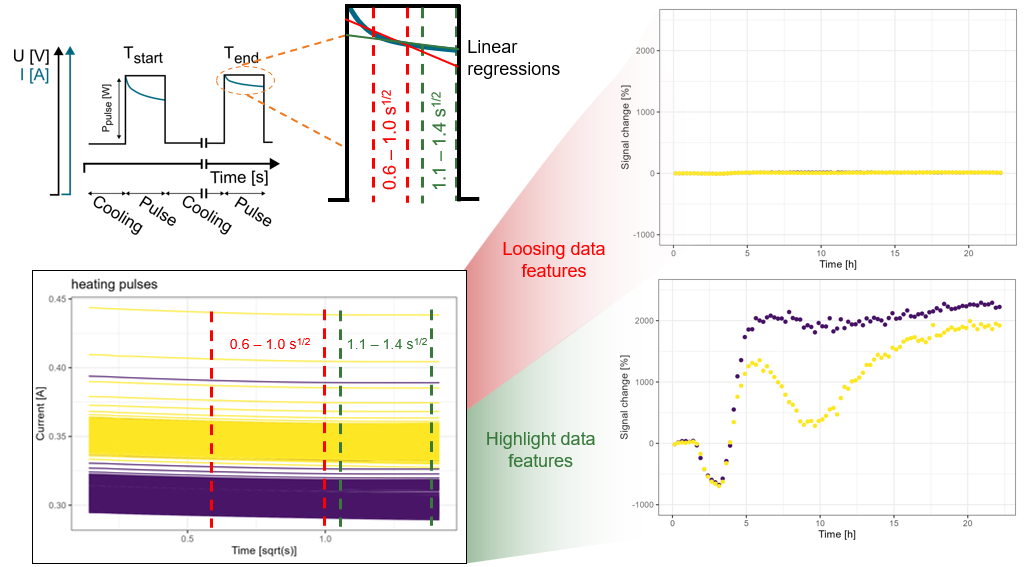


**Figure S6.** Modified Transient Plane Source (TPS) data analysis as reported in the literature. Traditionally, thermal response is analyzed by applying linear regression to a selected interval of the heating curve. Two different fitting intervals are shown here to exemplify how user selection impacts the resulting trends and thermal parameters. The significant variability between these fits reveals a major limitation of this method: it is highly sensitive to subjective interval choice and prone to errors from noise or signal artifacts. This motivated the development of a new data analysis approach based on full-curve fitting, enabling a more robust, consistent, and objective extraction of thermal properties. By leveraging the entire heating profile rather than a single segment, this method improves reliability and minimizes user-dependent variability.


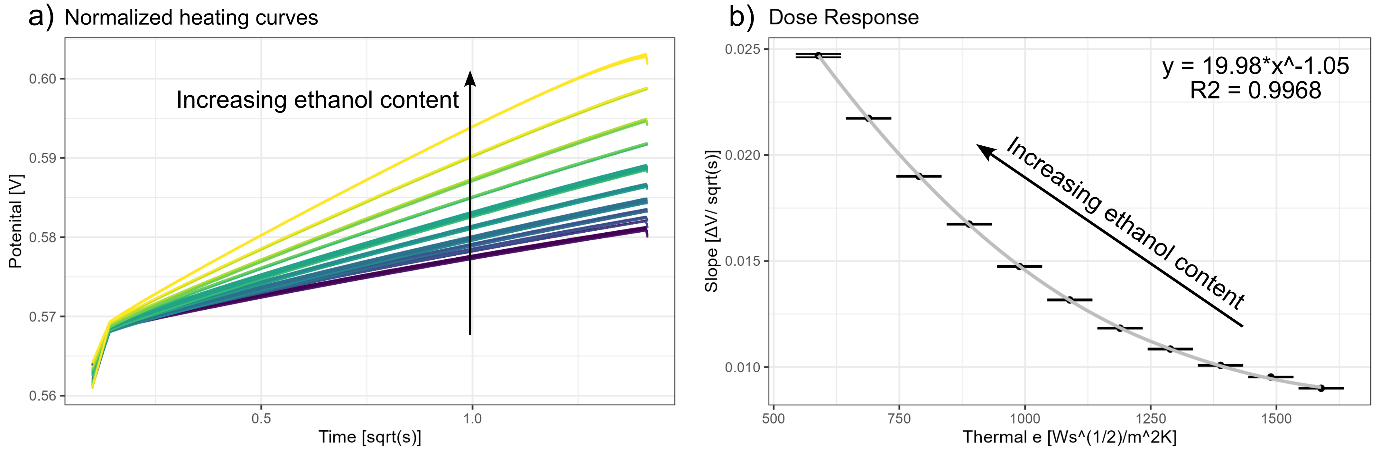


**Figure S7.** Verification experiment to test our in-house fabricated thermal sensors to the response to different thermal e. a) Normalized heating curves for various water-ethanol mixtures (0, 10, 20, 30, 40, 50, 60, 70, 80, and 90 vol%, and absolute ethanol). b) Corresponding dose-response curve (n = 10 per sample). The mean coefficient of variation is 0.188.


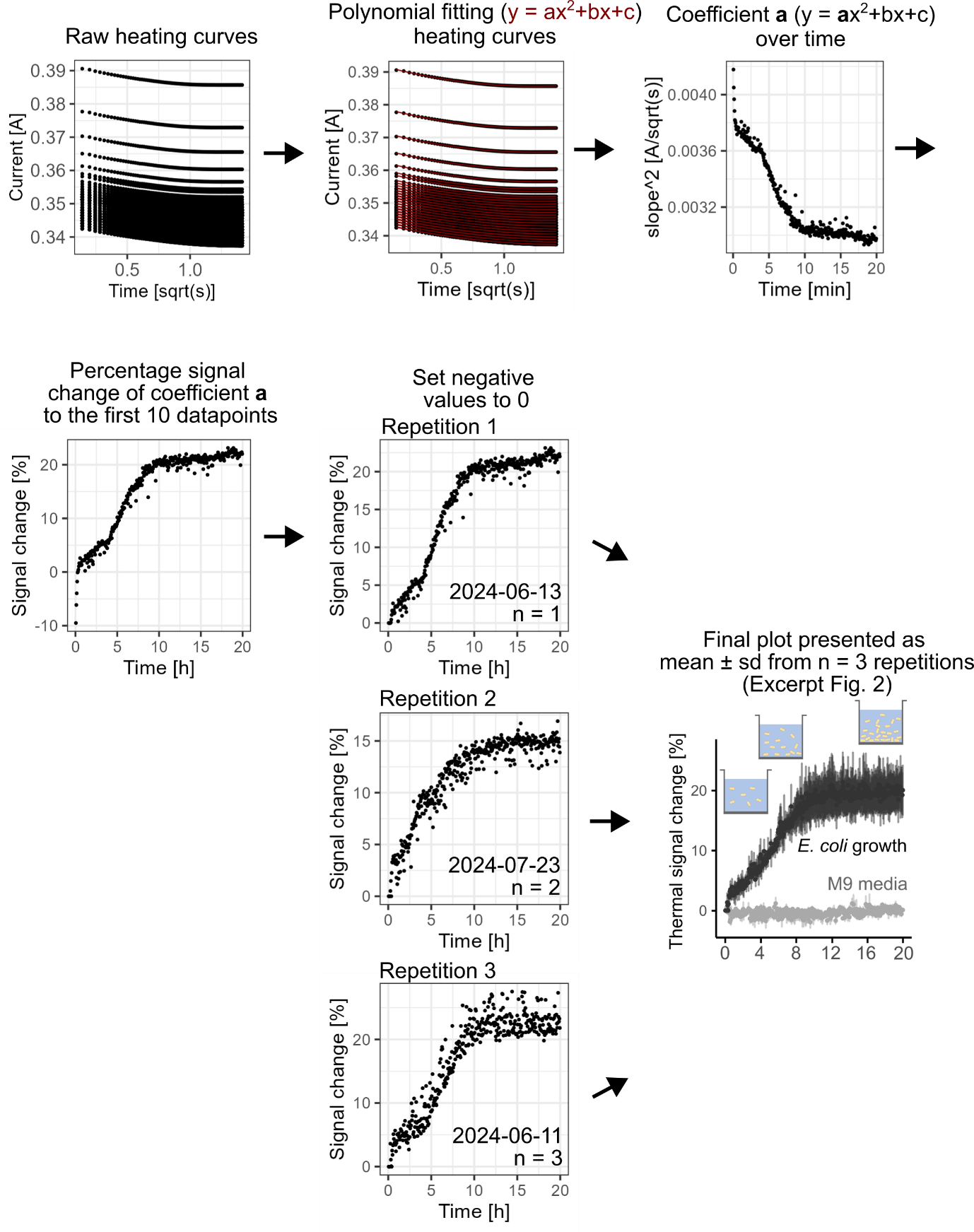


**Figure S8.** Step-by-step presentation of the data post-processing, including polynomial regression, feature extraction, and data transformation. Each experimental condition has been measured for n = 3 technical repetitions. The final plots are presented as mean ± sd (n=3).

**Table S1.** This table presents key parameters derived from growth curve analysis using the R package growthcurver. Fitting was performed on both optical density (OD) and thermal signal data, where applicable. The extracted metrics include the growth rate, carrying capacity, and time to mid-log phase, providing a comparative overview of bacterial growth dynamics under various CTX treatment conditions. These values highlight the differing kinetic responses captured by bulk (OD) versus interface-sensitive (thermal) measurements.

| Condition | k | n0 | r | t_mid | t_gen | auc_l | auc_e |
| --- | --- | --- | --- | --- | --- | --- | --- |
| OD_noCTX | 0,946603 | 0,113612 | 0,885219 | 2,250558 | 0,783023 | 17,37491 | 17,3915 |
| OD_0h_005ug | 0,856163 | 0,085811 | 1,166718 | 1,881088 | 0,5941 | 16,07737 | 16,11388 |
| OD_0h_1ug | 0,735142 | 0,085898 | 1,236083 | 1,636339 | 0,560761 | 13,97735 | 14,01063 |
| OD_1h_005ug | 0,871401 | 0,081144 | 1,191459 | 1,910372 | 0,581763 | 16,34538 | 16,38975 |
| OD_1h_1ug | 0,804883 | 0,085559 | 1,20644 | 1,764783 | 0,574539 | 15,2059 | 15,24088 |
| OD_2h_005ug | 0,903252 | 0,087036 | 1,082679 | 2,06743 | 0,640215 | 16,79053 | 16,82625 |
| OD_2h_1ug | 0,846418 | 0,082882 | 1,106057 | 2,007616 | 0,626683 | 15,78503 | 15,81538 |
| OD_3h_005ug | 0,924525 | 0,101114 | 0,927846 | 2,260302 | 0,74705 | 16,97878 | 16,99413 |
| OD_3h_1ug | 0,861864 | 0,077842 | 1,144349 | 2,018403 | 0,605713 | 16,07281 | 16,11913 |
| Thermal_noCTX | 0,452076 | 0,20333 | 0,259159 | 0,777914 | 2,674598 | 7,659647 | 7,651273 |
| Thermal_0h_005ug | 0,405393 | 0,240238 | 0,2156 | -1,73816 | 3,214969 | 7,141279 | 7,129146 |
| Thermal _0h_1ug | 0,744952 | 0,150801 | 0,442642 | 3,097693 | 1,565932 | 12,21169 | 12,20734 |
| Thermal_1h_005ug | 0,331743 | 0,253936 | 0,220677 | -5,36008 | 3,140999 | 6,238627 | 6,226439 |
| Thermal _1h_1ug | 1,280582 | 0,173918 | 0,096959 | 19,0856 | 7,148872 | 7,825347 | 7,823675 |
| Thermal_2h_005ug | 0,354162 | 0,238833 | 0,265724 | -2,73958 | 2,608523 | 6,561291 | 6,549846 |
| Thermal _2h_1ug | 0,471505 | 0,219796 | 0,121696 | 1,114046 | 5,695713 | 6,843783 | 6,833259 |
| Thermal_3h_005ug | 0,281527 | 0,235668 | 0,233913 | -6,99766 | 2,963274 | 5,418725 | 5,407582 |
| Thermal _3h_1ug | 0,889244 | 0,180807 | 0,071934 | 18,98442 | 9,635812 | 6,218415 | 6,214504 |

**Table S2.** Summary of all experimental conditions used throughout the study, including the date of each experiment, specific CTX treatment conditions, the number of repetitions performed for statistical robustness, and the corresponding SMU channels and thermal interfaces utilized. This documentation ensures reproducibility and provides a clear overview of the experimental design across different biological scenarios.

| Experimental condition | | | | |  |  |  |
| --- | --- | --- | --- | --- | --- | --- | --- |
| Date of experiment [YYYY-MM-DD] | Mircobe | Antibiotic - CTX | Concentration [µg/mL] | Time of addition [h] | Repetition | SMU channel | Sensor ID |
| 2024-06-13 | *E. coli* YFP | No | - | - | 1 | 1 | S1 |
| 2024-07-23 | *E. coli* YFP | No | - | - | 2 | 1 | S3 |
| 2024-06-11 | *E. coli* YFP | No | - | - | 3 | 2 | S2 |
| 2024-10-01 | *E. coli* YFP | Yes | 0.05 | 0 | 1 | 1 | S2 |
| 2024-10-09 | *E. coli* YFP | Yes | 0.05 | 0 | 2 | 2 | S2 |
| 2024-10-09 | *E. coli* YFP | Yes | 0.05 | 0 | 3 | 1 | S4 |
| 2024-07-17 | *E. coli* YFP | Yes | 1 | 0 | 1 | 1 | S4 |
| 2024-07-17 | *E. coli* YFP | Yes | 1 | 0 | 2 | 2 | S3 |
| 2024-09-10 | *E. coli* YFP | Yes | 1 | 0 | 3 | 1 | S2 |
| 2024-09-23 | *E. coli* YFP | Yes | 10 | 0 | 1 | 1 | S4 |
| 2024-09-23 | *E. coli* YFP | Yes | 10 | 0 | 2 | 2 | S4 |
| 2024-09-26 | *E. coli* YFP | Yes | 10 | 0 | 3 | 2 | S2 |
| 2024-09-04 | *E. coli* YFP | Yes | 250 | 0 | 1 | 1 | S3 |
| 2024-09-04 | *E. coli* YFP | Yes | 250 | 0 | 2 | 2 | S4 |
| 2024-09-11 | *E. coli* YFP | Yes | 250 | 0 | 3 | 1 | S2 |
| 2024-06-26 | *E. coli* YFP | Yes | 500 | 0 | 1 | 1 | S1 |
| 2024-07-22 | *E. coli* YFP | Yes | 500 | 0 | 2 | 1 | S3 |
| 2024-07-22 | *E. coli* YFP | Yes | 500 | 0 | 3 | 2 | S4 |
| 2024-10-08 | *E. coli* YFP | Yes | 0.05 | 1 | 1 | 1 | S4 |
| 2024-10-24 | *E. coli* YFP | Yes | 0.05 | 1 | 2 | 1 | S2 |
| 2024-10-24 | *E. coli* YFP | Yes | 0.05 | 1 | 3 | 2 | S4 |
| 2024-07-11 | *E. coli* YFP | Yes | 1 | 1 | 1 | 1 | S3 |
| 2024-07-11 | *E. coli* YFP | Yes | 1 | 1 | 2 | 2 | S4 |
| 2024-09-13 | *E. coli* YFP | Yes | 1 | 1 | 3 | 2 | S2 |
| 2024-09-25 | *E. coli* YFP | Yes | 10 | 1 | 1 | 1 | S4 |
| 2024-09-25 | *E. coli* YFP | Yes | 10 | 1 | 2 | 1 | S2 |
| 2024-10-07 | *E. coli* YFP | Yes | 10 | 1 | 3 | 2 | S2 |
| 2024-09-17 | *E. coli* YFP | Yes | 250 | 1 | 1 | 1 | S4 |
| 2024-09-05 | *E. coli* YFP | Yes | 250 | 1 | 2 | 2 | S4 |
| 2024-09-17 | *E. coli* YFP | Yes | 250 | 1 | 3 | 2 | S2 |
| 2024-07-16 | *E. coli* YFP | Yes | 500 | 1 | 1 | 2 | S3 |
| 2024-09-18 | *E. coli* YFP | Yes | 500 | 1 | 2 | 1 | S4 |
| 2024-09-18 | *E. coli* YFP | Yes | 500 | 1 | 3 | 2 | S2 |
| 2024-11-28 | *E. coli* YFP | Yes | 0.05 | 2 | 1 | 1 | S4 |
| 2024-12-04 | *E. coli* YFP | Yes | 0.05 | 2 | 2 | 1 | S2 |
| 2024-12-04 | *E. coli* YFP | Yes | 0.05 | 2 | 3 | 2 | S4 |
| 2024-11-29 | *E. coli* YFP | Yes | 1 | 2 | 1 | 1 | S4 |
| 2024-11-29 | *E. coli* YFP | Yes | 1 | 2 | 2 | 2 | S2 |
| 2024-12-05 | *E. coli* YFP | Yes | 1 | 2 | 3 | 2 | S4 |
| 2024-12-02 | *E. coli* YFP | Yes | 10 | 2 | 1 | 2 | S4 |
| 2024-12-06 | *E. coli* YFP | Yes | 10 | 2 | 2 | 1 | S4 |
| 2024-12-06 | *E. coli* YFP | Yes | 10 | 2 | 3 | 2 | S2 |
| 2025-01-28 | *E. coli* YFP | Yes | 250 | 2 | 1 | 1 | S4 |
| 2025-01-15 | *E. coli* YFP | Yes | 250 | 2 | 2 | 1 | S2 |
| 2025-01-15 | *E. coli* YFP | Yes | 250 | 2 | 3 | 2 | S4 |
| 2024-12-10 | *E. coli* YFP | Yes | 500 | 2 | 1 | 1 | S4 |
| 2024-12-10 | *E. coli* YFP | Yes | 500 | 2 | 2 | 2 | S3 |
| 2025-01-06 | *E. coli* YFP | Yes | 500 | 2 | 3 | 1 | S2 |
| 2024-10-15 | *E. coli* YFP | Yes | 0.05 | 3 | 1 | 1 | S2 |
| 2024-10-22 | *E. coli* YFP | Yes | 0.05 | 3 | 2 | 1 | S4 |
| 2024-10-22 | *E. coli* YFP | Yes | 0.05 | 3 | 3 | 2 | S2 |
| 2024-07-15 | *E. coli* YFP | Yes | 1 | 3 | 1 | 1 | S4 |
| 2024-07-15 | *E. coli* YFP | Yes | 1 | 3 | 2 | 2 | S3 |
| 2024-09-12 | *E. coli* YFP | Yes | 1 | 3 | 3 | 1 | S2 |
| 2024-09-24 | *E. coli* YFP | Yes | 10 | 3 | 1 | 1 | S2 |
| 2024-09-24 | *E. coli* YFP | Yes | 10 | 3 | 2 | 2 | S4 |
| 2024-09-30 | *E. coli* YFP | Yes | 10 | 3 | 3 | 1 | S2 |
| 2024-09-03 | *E. coli* YFP | Yes | 250 | 3 | 1 | 1 | S3 |
| 2024-09-03 | *E. coli* YFP | Yes | 250 | 3 | 2 | 2 | S4 |
| 2024-09-16 | *E. coli* YFP | Yes | 250 | 3 | 3 | 2 | S2 |
| 2024-06-27 | *E. coli* YFP | Yes | 500 | 3 | 1 | 1 | S1 |
| 2024-06-27 | *E. coli* YFP | Yes | 500 | 3 | 2 | 2 | S2 |
| 2025-01-28 | *E. coli* YFP | Yes | 500 | 3 | 3 | 1 | S4 |

**Table S3.** This table presents the calculated CTX/bacteria ratios used to account for both antibiotic concentration and the time point of CTX administration. Since bacterial cultures grow over time, a later addition of CTX effectively corresponds to a higher bacterial load at the moment of exposure. Therefore, this ratio serves as a valuable metric to represent the combined effect of concentration and bacterial density in this bi-parametric study. The observed signal trends in Case 3 and Case 4 correlate with these ratios, supporting their relevance in interpreting the antibiotic-induced bacterial responses.

|  |  | CTX concentration [µg/mL] | | | | |
| --- | --- | --- | --- | --- | --- | --- |
| CTX addition timepoint [h] | Corresponding OD_600_ values after [h] of incubation | 0.05 | 1 | 10 | 250 | 500 |
| 0 | 0.143 | 0.349 | 6.993 | 69.930 | 1748.252 | 3496.504 |
| 1 | 0.240 | 0.208 | 4.167 | 41.167 | 1041.667 | 2083.334 |
| 2 | 0.420 | 0.119 | 2.381 | 23.810 | 595.238 | 1190.476 |
| 3 | 0.600 | 0.083 | 1.667 | 16.667 | 416.667 | 833.334 |

**Table S4**. This table converts the absolute OD600 values after 20 h of incubation into Δ relative absorbance unit (RAU). For the calculation, the background (M9 media) was subtracted from the RAU at the given time point. The table reports mean ± sd (n=3).

| **Condition** | **Abs. OD600 after 20h** | **ΔRAU after 20h** |
| --- | --- | --- |
| **Background:** | |  |
| M9 media | 0.05 ± 0.00 |  |
| **Case 0** |  |  |
| no CTX addition | 0.89 ± 0.04 | 0.84 ± 0.04 |
| **Case 1** |  |  |
| 0.05 µg/mL at t = 0h | 0.75 ± 0.08 | 0.70 ± 0.08 |
| 0.05 µg/mL at t = 1h | 0.72 ± 0.08 | 0.67 ± 0.08 |
| 0.05 µg/mL at t = 2h | 0.82 ± 0.05 | 0.77 ± 0.05 |
| 0.05 µg/mL at t = 3h | 0.92 ± 0.01 | 0.87 ± 0.01 |
| 1 µg/mL at t = 0h | 0.61 ± 0.05 | 0.56 ± 0.05 |
| 1 µg/mL at t = 1h | 0.71 ± 0.02 | 0.66 ± 0.02 |
| 1 µg/mL at t = 2h | 0.81 ± 0.03 | 0.76 ± 0.03 |
| 1 µg/mL at t = 3h | 0.72 ± 0.06 | 0.67 ± 0.06 |
| **Case 2** | |  |
| 250 µg/mL at t = 0h | 0.07 ± 0.01 | 0.02 ± 0.01 |
| 250 µg/mL at t = 1h | 0.07 ± 0.01 | 0.02 ± 0.01 |
| 500 µg/mL at t = 0h | 0.06 ± 0.01 | 0.01 ± 0.01 |
| 500 µg/mL at t = 1h | 0.06 ± 0.01 | 0.01 ± 0.01 |
| **Case 3** | | |
| 10 µg/mL at t = 0h | 0.07 ± 0.01 | 0.02 ± 0.01 |
| 10 µg/mL at t = 1h | 0.08 ± 0.01 | 0.03 ± 0.01 |
| 250 µg/mL at t = 2h | 0.10 ± 0.02 | 0.05 ± 0.02 |
| 500 µg/mL at t = 2h | 0.10 ± 0.04 | 0.05 ± 0.04 |
| **Case 4** | | |
| 10 µg/mL at t = 2h | 0.13 ± 0.02 | 0.08 ± 0.02 |
| 10 µg/mL at t = 3h | 0.34 ± 0.04 | 0.29 ± 0.04 |
| 250 µg/mL at t = 3h | 0.16 ± 0.06 | 0.11 ± 0.06 |
| 500 µg/mL at t = 3h | 0.16 ± 0.07 | 0.11 ± 0.07 |
